# In-Depth Understanding of Granule Compression Behavior under Variable Raw Material and Processing Conditions

**DOI:** 10.3390/pharmaceutics14010177

**Published:** 2022-01-12

**Authors:** Tibor Casian, Sonia Iurian, Alexandru Gâvan, Alina Porfire, Anca Lucia Pop, Simona Crișan, Anda Maria Pușcaș, Ioan Tomuță

**Affiliations:** 1 Department of Pharmaceutical Technology and Biopharmacy, Faculty of Pharmacy, “Iuliu Hațieganu” University of Medicine and Pharmacy, 400000 Cluj-Napoca, Romania; casian.tibor@umfcluj.ro (T.C.); gavan.alexandru@umfcluj.ro (A.G.); aporfire@umfcluj.ro (A.P.); andapsc9@gmail.com (A.M.P.); tomutaioan@umfcluj.ro (I.T.); 2 Department of Clinical Laboratory, Faculty of Pharmacy, “Carol Davila” University of Medicine and Pharmacy, 050474 Bucharest, Romania; anca.pop@umfcd.ro; 3 RD Center, AC HELCOR, 430092 Baia Mare, Romania; simonacrisan@achelcor.ro

**Keywords:** dynamic compaction analysis, granulation, design space, raw material variability, microcrystalline cellulose

## Abstract

Tablet manufacturing involves the processing of raw materials through several unit operations. Thus, the mitigation of input-induced variability should also consider the downstream processability of intermediary products. The objective of the present work was to study the effect of variable raw materials and processing conditions on the compression properties of granules containing two active pharmaceutical ingredients (APIs) and microcrystalline cellulose. Differences in compressibility and tabletability of granules were highlighted in function of the initial particle size of the first API, granule polydispersity and fragmentation. Moreover, interactions were underlined with the atomizing pressure. Changing the supplier of the second API was efficiently controlled by adapting the binder addition rate and atomizing pressure during granulation, considering the starting crystal size. By fitting mathematical models on the available compression data, the influence of diluent source on granule compactibility and tabletability was identified. These differences resumed to the ease of compaction, tableting capacity and pressure sensitivity index due to variable water binding capacity of microcrystalline cellulose. Building the design space enabled the identification of suitable API types and the appropriate processing conditions (spray rate, atomizing pressure, compression force) required to ensure the desired tableting performance.

## 1. Introduction

Tablet manufacturing requires the processing of raw materials through several unit operations. Thus, the mitigation of input-induced variability in intermediate product characteristics should also consider downstream processability during subsequent unit operations. To ensure process robustness and have in-depth product knowledge, it is recommended to consider both raw material- and process-related factors from the critical unit operations. In this case, input variability can be managed accordingly to ensure a reproducible performance during the various processing steps and for the final product [1].

Due to cost- and time-related reasons, raw material variability is usually not rigorously considered during pharmaceutical development. However, raw material manufacturers may present slight modifications in the synthesis and crystallization of active pharmaceutical ingredients (API) or the quality of natural products used for excipient production during the product’s lifecycle. In some situations, even minor changes can significantly impact the processability of the materials [2,3].

Reaching a robust formulation can be a challenge, especially for highly dosed products, where the API characteristics will largely contribute to the overall processing performance. In such cases, thorough investigations are needed to reduce the impact of input variability by optimizing the formulation and the process [4].

Building the design space for complex unit operations considering different raw material suppliers provides the opportunity of a well-controlled environment with the possibility of science-based adjustment of critical process parameters [5]. According to ICH Q8, the process of gaining enhanced product-related knowledge should revolve around an investigated domain of raw material attributes, processing parameters and manufacturing options. Fonteyne et al. applied an experimental design-based approach to identify the required process adjustments to reach the target granule size distribution from microcrystalline cellulose (MCC) samples with different water binding capacity [6]. Stauffer et al. built the design space for a continuous granulation process by incorporating into the study API-related variability, thus controlling the risk of failure coming from this source. A 1% risk of failure could be achieved by specifying limits for the size of fine fraction, span and granule friability [7]. Hwang et al. demonstrated the importance of incorporating raw material- and process-related factors when a new formulation is being developed or during technological transfer. The study investigated diluents of different grades in the context of twin-screw granulation [8]. Using a DoE approach, Matsunami et al. showed how the granule/tablet performance and the maximum acceptable manufacturing rate are linked to the applied liquid-solid ratio and granule circularity for a continuous high shear granulation process [9].

Identifying critical parameters that impact product quality and the overall productivity of a manufacturing line can reduce manufacturing costs while maintaining the assumed quality level. Developing a link between processing conditions, input variability and product performance requires the use of specialized instruments such as design of experiments and multivariate data analysis that allow the calculation of design space [3,10,11,12,13,14]. Such an approach can lead to an extended root cause analysis [3] and the implementation of feed-forward process control methods [15]. 

Formulation design should include excipient type, grade and amount selection based on understanding the granulation process and tableting properties. However, frequently, this process relies on empirical rules and formulator experience [16,17]. Additionally, most studies that focus on estimating the impact of variable raw materials are oriented primarily on one component-related variability, be it the API [7] or the diluent [18]. In the case of more complex formulations, presenting multiple large dose APIs and diluents, inter-component interactions combined with process variability can convolute into a less predictable and more difficult to manage process [7].

Pharmaceutical granulation was described as a highly unrepeatable processing step concerning current draw and several output-related variables, even under well-controlled experimental conditions [19]. As granulation is often applied to increase the materials’ flowability and tabletability, the difficult to process high dose formulations require this unit operation [20,21]. Using historical data, we demonstrated how variable material attributes and differences in processing could impact granule downstream processability and tablet disintegration. The study highlighted the importance of raw material management and feed-forward process control models [15].

As a result of input variability, the agglomeration stage of granulation can be highly variable, leading to granules with different size distribution and flow properties. The effect of particle size on compactibility and compressibility is material- and formulation-dependent. Size may not impact the densification under compaction (compressibility) for highly fragmenting granules. In contrast, the compactibility will be influenced by the size and fragmenting behavior as they strongly relate to the inter-particulate bonding surface area [22]. Several studies have shown how factors such as the nature of the binder [23], the intragranular porosity [24], particle size and diluent surface area can influence the ability of the granulation method of producing an intermediate product with increased tensile strength and lower solid fractions [25]. Considering the multitude of factors and possible interactions, an in-depth evaluation is required for each developed product. 

Compaction simulators have been proven beneficial for investigating tableting or other upstream unit operations’ processing variables, identifying scale-up parameters, predicting optimal tableting conditions when the tableting machine is changed, troubleshooting, guiding formulation development and reducing tablet failures [16,26]. Dynamic compaction analysis offers the opportunity to evaluate the compression behavior of multi-particulate systems to provide an improved level of understanding regarding the influence of raw material-, formulation- and process-related factors [16,17,27,28,29]. 

The objective of the present work was to study the effect of variable raw materials and processing conditions on the tableting properties of granules prepared by fluid bed granulation of two APIs and a diluent. The main elements of novelty are represented by the simultaneous investigation of multiple sources of variability and the approach used to obtain robust downstream processing performance of granules. 

To reach an in-depth understanding of granule compression behavior, the investigation was structured as follows: (1) application of Design of Experiments to describe the impact of raw material and process variations on granule properties; (2) application of multivariate tools to describe the effect of variable input and granule size descriptors on CCT profiles (compressibility, compactability, tabletability); (3) mathematical modeling of CCT profiles and use of multivariate tools to provide an overview on granule performance variability; (4) building the design space. 

## 2. Materials and Methods

### 2.1. Materials

For the preparation of granules, two APIs, a diluent and a binder were used in the formulation. To this respect, the APIs were acquired from three different suppliers and were coded as IBU A-B-C for ibuprofen and PAR A-B-C for paracetamol. Microcrystalline cellulose was supplied from two different producers and was coded as MCC A-B. The binder, hydroxypropyl methylcellulose—Methocel E5LV, was kindly donated by Colorcon (Dartford, UK). Due to confidentiality aspects, the source of raw materials was not divulged. 

### 2.2. X-Ray Powder Diffraction (XRPD)

XRPD diffractograms were acquired using a Bruker D8 Advanced X-ray diffractometer (Bruker AXS, Karlsruhe, Germany), equipped with a LynxEye super speed detector and with a Ge (1 1 1) filtered Cu Kα1 radiation source. The measurements were conducted in the angular 2θ between 3–40 degrees.

### 2.3. Scanning Electron Microscopy (SEM)

SEM micrographs were recorded on the API samples coming from different suppliers. Samples were coated with Pt/Pd using an Agar Automatic Sputter coater (Agar Scientific, Stansted, Essex, UK) followed by image acquisition with a Quanta 3D FEG equipment (FEI, Thermo Scientific, Dreiech, Germany at 15 kV.

### 2.4. Water Binding Capacity

Following, 5 g of MCC was suspended in 20 mL of purified water followed by centrifugation at 5000 *g* for 20 min (Sigma 3K30 centrifuge, Darmstadt, Germany). The MCC pellet was weighed after eliminating the supernatant, and the water binding capacity was calculated as the relative percentage of water uptake with respect to the initial sample mass. 

### 2.5. Bulk and Tapped Density

A sample size of 100 g was loaded into a cylinder, and the bulk density was calculated as the ratio between the mass and initial volume. Following a series of taps, the final volume was determined and used to calculate the tapped density. To this respect, the volume was recorded after 750 and 1250 taps, whereas if the difference exceeded 2 mL, another 500 taps were performed to determine the tapped volume. Experiments were performed using an Erweka SVM 100 (Erweka, Langen, Germany) powder density tester. 

### 2.6. Loss on Drying

The moisture content of MCC samples was evaluated using a thermogravimetric moisture analyzer (Mettler Toledo, Columbus, OH, USA) and by calculating the percentage of weight loss from samples exposed to a temperature of 100 °C for 10 min.

### 2.7. Granule Preparation

The granule preparation was done using a lab-scale fluidized bed granulator (Aeromatic Strea 1, GEA, Dusseldorf, Germany) by processing 200 g sized batches. The formulations were prepared according to a D-optimal experimental design with 39 runs (Supplementary Materials, Table S1). All the formulations presented identical quantitative composition, whereas the source of raw materials, the atomizing pressure (0.5–0.75 atm) and the spraying rate were varied in a controlled manner, as suggested by the experimental design matrix (Table 1). The quantitative composition involved: 40.22% IBU; 32.68% PAR; 23.79% MCC and 3.31% HPMC. Initially, the components were loaded into the expansion vessel of the granulator, the loading order being identical for all the formulations. This step was followed by a pre-heating and mixing step of 10 min, through an applied airflow of 3–4.5 m^3^/min and an inlet air heated to 30 °C. The next step included the addition of a 10% (*m*/*m*) binder solution with a pre-defined rate and atomizing pressure through a 0.8 mm nozzle. Finally, a drying stage was conducted in the same device by maintaining the fluidization for another period of 10 min at 40 °C.

### 2.8. Particle Size Analysis

Granulometric analysis was performed on 100 g of each granule formulation, using a sieve system (Retsch, Dusseldorf, Germany) that included 9 sieves of 100, 200, 300, 400, 500, 600, 710, 800 and 900 µm, covering a size range of 0–1000 μm. Each size fraction was weighed, followed by the calculation of central tendency (average particle size, Xa) and dispersion index descriptors (Span). In the case of raw materials, the particle size was measured from SEM micrographs using Image J software.

### 2.9. Dynamic Compaction Analysis

Dynamic compaction analysis was performed using a single punch Gamlen GTP, series D tablet Press (Gamlen Tableting Ltd., Biocity Nottingham, Nottingham, UK) to evaluate the impact of variables on the compression behavior of granules; 100 mg sized compacts were prepared from each formulation, using 6 mm punches at a 10 mm/min speed and five different loads (100 kg–200 kg–300 kg–400 kg–500 kg). The obtained force-displacement curves from the compaction, detachment and ejection steps were used to calculate several performance indicators (work of compression, elastic recovery, detachment stress (DS), ejection stress (ES), solid fraction). Each compact was measured in diameter, thickness and hardness to calculate the compact tensile strength (TS). The obtained results were used as responses in an extended experimental design (Supplementary Materials Table S1). Additionally, the results were visualized by plotting compressibility, compactibility and tabletability profiles. 

Different mathematical models were considered after the dynamic compaction analysis to interpret the granule compression behavior. To this respect, the Heckel equation (1) was used to evaluate the compressibility, the Ryshkewitch–Duckworth (2) model for compactibility and the Power model (3) for tabletability. 

The mean yield pressure, calculated from the porosity pressure relationship (Heckel equation), was used to evaluate the degree of plastic deformation and to classify the materials. The mean yield pressure (Py) was calculated as the reciprocal of the slope. According to the compression behavior classification system proposed by Dai et al., the product can be very soft (Py < 40); soft (Py ∈ (40, 80)); moderately hard (Py ∈ (80, 200)) and hard (Py > 200) [30].

ln 1/ε = kP + A(1)

where ε—bed porosity; P—compression pressure; k, A—constants.

The correlation between TS and porosity, described by the Ryshkewitch–Duckworth equation, was used to evaluate the bonding capacity between the particles. The k_b_ parameter was calculated and used to classify the formulations as easily compacted (k_b_ < 10) and difficulty compacted (k_b_ > 10) products [30].

TS = T_0_ exp(−k_b_ ε) (2)

where TS—tensile strength, T0—tensile strength at zero porosity; k_b_—constant; ε—porosity.

The tabletability capacity (d) and a pressure sensitivity index (g) were calculated by fitting a power model on the TS and compression pressure profiles. Based on the d parameter, tabletability was classified as unacceptable (d < 2 × 10^−3^), excellent (d ≥ 0.5) and intermediate (2 × 10^−3^ < d < 0.5) [30].

TS = dP^g^ (3)

where TS—tensile strength; P—compression pressure; d—tabletability capacity; g—pressure sensitivity index.

### 2.10. Design of Experiments: Effect Analysis and Design Space Estimation

The previous experimental design was extended to identify the influence of input factors on the granule compression behavior by introducing the compression force as a quantitative factor with five levels of variation. The initial design was multiplied using five blocks. The R2 parameter was calculated for each response to evaluate the percentage of response variation explained through input variation. The Q2 parameter reflecting the fraction from the total response variation that can be predicted by the model was calculated using the principles of cross-validation. The effect of input factors was analyzed by generating coefficient plots, revealing the exerted effect’s significance, magnitude and direction. For each coefficient, an error bar was represented, showing the significance of the respective term. Interactions were analyzed by representing interaction plots. 

The design space or the factor combination range that delivers the desired quality profile was computed to identify a robust formulation and select the suppliers accordingly. Using the fitted polynomial models and Monte Carlo simulations, the experimental region was mapped in terms of probability of failure, expressed as a percentage (%) [31]. The acceptance limit for the probability of failure was set to 1%. 

This data analysis section was done using Modde Pro 12.1 (Sartorius Stedim Biotech, Göttingen, Germany).

### 2.11. Multivariate Data Analysis: PCA, O2PLS, OPLS-DA

O2PLS analysis was applied to provide a numerical estimation of performance variability as a result of input variation. Developing such models made it possible to quantify the amount of variation in granule characteristics caused by changing the supplier of the used raw materials. Separate models were built for granule compressibility, compactibility and tabletability. The X (DoE matrix; granule size descriptors) and Y matrix (granule compressibility, compactibility and tabletability) were scaled to unit variance. Model interpretation included the estimation of unique and joint sources of variation and the identification of correlated input variables through the generation of loading plots [3]. 

To provide an extended root cause analysis, a PCA model was computed on the compression parameters calculated by fitting mathematical models on dynamic compaction analysis data [32]. Thus, the k_H_ (Heckel), k_b_ (Ryshkwitch–Duckworth) and the d and g parameters (Power model) were initially scaled to unit variance. Hierarchical cluster analysis was performed on the latent variables of the PCA model, enabling the classification of formulations considering the combination pattern of the compression parameters. Discriminant analysis (OPLS-DA) was used to highlight the differences between classes [33]. Biplots were represented for better visualization of the relationship between observations and variables. Multivariate data analysis was performed using SIMCA 15 (Sartorius Stedim Biotech, Göttingen, Germany).

## 3. Results and Discussion

### 3.1. The Influence of Input Variables on Granule Characteristics

Before proceeding to model interpretation, the fitted models were evaluated in terms of R2, Q2, Validity, Reproducibility, ANOVA for regression significance and lack of fit. A fine-tuning step was included for each response by excluding non-significant terms and evaluating the evolution of model statistics. For all characteristics, the response variation could be explained accordingly by the independent variables (R2 > 0.835) and predicted with good predictive capacity (Q2 > 0.776). Details on the obtained model performance results are included in Table 2.

#### 3.1.1. Average Particle Size (Xa) and Size Distribution (Span)

**Figure 1:**
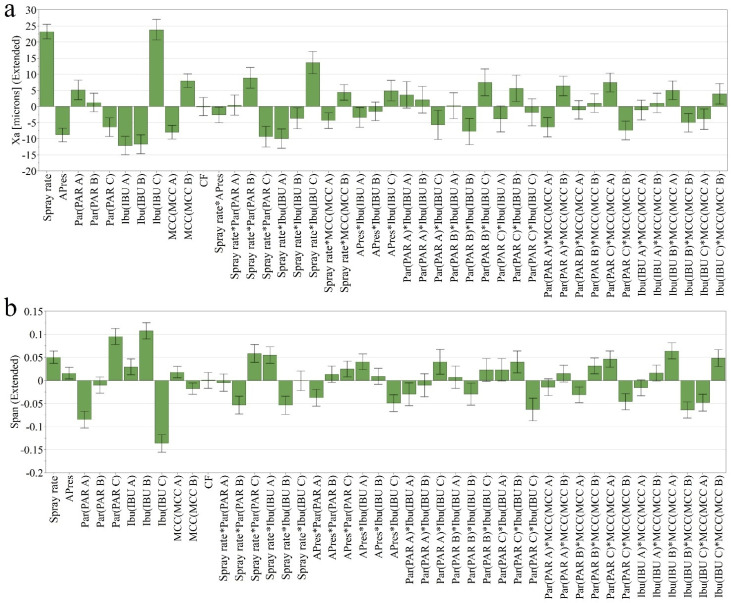
Coefficient plots presenting input variables’ influence on Xa (**a**) and span (**b**).

The average particle size and the distribution of granules were influenced by both process factors and raw materials. The obtained formulations had the Xa ranging between 214 µm and 387 µm, with an Avg. ± SD of 289 ± 35 µm (Supplementary Materials Table S2). Higher binder addition rates and lower atomizing pressures lead to better wetting of the powder particles, contributing to increased particle growth during the granulation process (Figure 1a). These process factors also presented significant interaction terms with different types of raw materials, namely PAR, IBU and MCC. Among the raw materials, IBU C had the most important influence on this response, the use of this type leading to granules with increased particle size.

**Figure 2:**
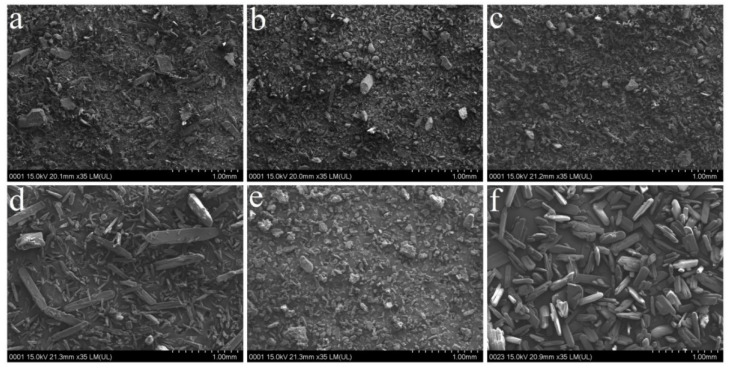
SEM micrographs of IBU and PAR types: PAR A (**a**); PAR B (**b**); PAR C (**c**); IBUA (**d**); IBUB (**e**); IBUC (**f**).

**Figure 3:**
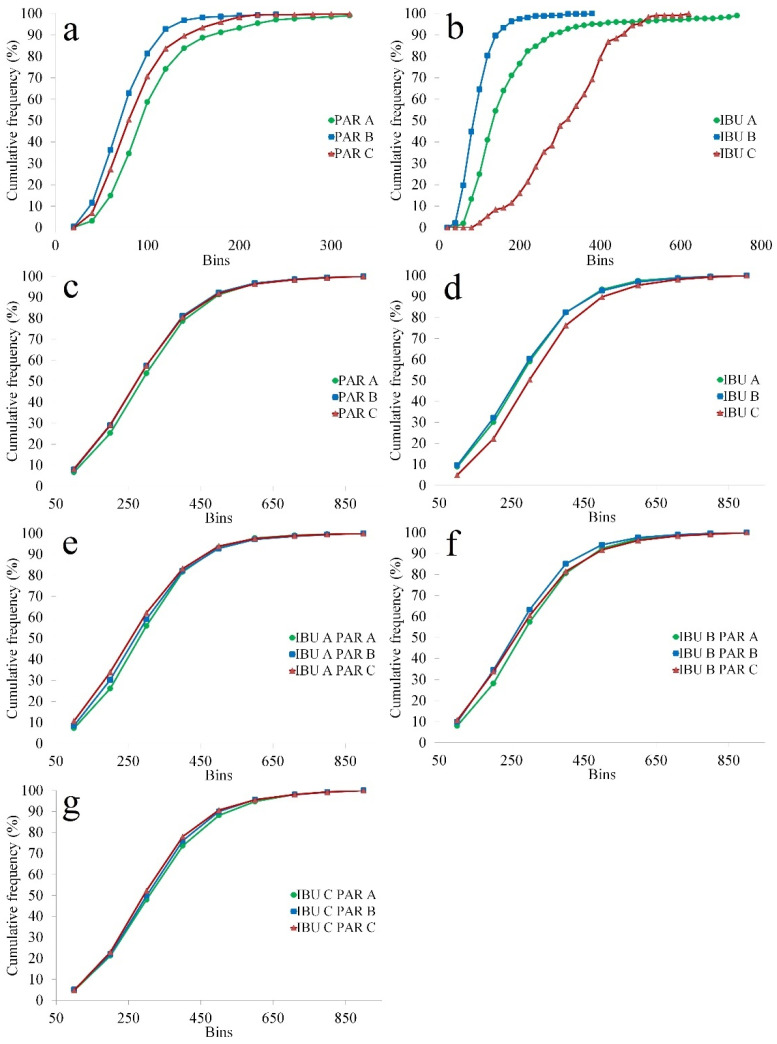
Particle size distribution of (**a**) PAR types; (**b**) IBU types; (**c**) Granules classified in function of PAR type (avg. profiles); (**d**) Granules classified in function of IBU type (avg. profiles); (**e**) IBU A- based granules classified in function of PAR type (avg. profiles); (**f**) IBU B-based granules classified in function of PAR type (avg. profiles); (**g**) IBU C-based granules classified in function of PAR type (avg. profiles).

The effect of raw materials on granule particle size and distribution could be explained by the initial particle size of API particles. Generally, granulating raw materials with larger initial particle sizes yielded larger granules with a more uniform distribution. SEM micrographs and particle size distribution for the investigated API types are presented in Figure 2a–f and Figure 3a,b.

Having granules with a narrow distribution from the mean is essential to avoid segregation and ensure appropriate flow properties during the subsequent compression step, which grants the mass and content uniformity of the prepared tablets [21]. The span values ranged between 1.2 and 2.1, with an Avg. ± SD of 1.6 ± 0.2. Low span values were obtained using low spray rate and atomizing pressure, PAR A and IBU C, depending on their combination with other factors (Figure 1b). For example, granules prepared using IBU C and PAR A were distributed in a slightly larger interval, whereas IBU C with PAR C showed a narrow distribution. Additionally, in the case of MCC, an interaction was identified with the source of APIs. 

For IBU, the initial particle size varied in the following order: IBU C (311 ± 110 µm) > IBU A (170 ± 131 µm) > IBU B (94 ± 43 µm). Interestingly, the size difference between A and B types did not impact the granule size, as these factors had similarly sized coefficients. In the case of type C, the particles of IBU were sufficiently large to act as agglomeration centers during granulation, thus favoring granule growth. Particle agglomeration during granulation is achieved if the desired level of liquid saturation is obtained, being influenced by the initial particle size of input materials. Due to the larger surface area associated with smaller particles, larger amounts of liquid are required [34]. Regarding the impact of granule size distribution, the observed effects were in accordance with IBU particle size. The highest span values were obtained for IBU B, intermediate for IBU A and lowest for IBU C. 

The particle size of PAR decreased from PAR A (104 ± 56 µm) to PAR C (88 ± 42 µm) and PAR B (75 ± 35 µm). In the case of PAR A, due to the larger initial particles, a positive influence was observed on granule size and a negative effect on the span value. Although the particle size difference between PAR B and C was small, granules prepared using type C had larger span values.

In the case of PAR, the calculated coefficients were smaller compared to IBU due to the lower proportion of this ingredient in the formulation and the smaller initial particles of this API. This observation is also confirmed by the average cumulative particle size distributions calculated on formulations prepared using different sources of PAR (Figure 3c) or IBU (Figure 3d). The lower cumulative frequencies from the initial part of the distribution associated with granules containing PAR A are maintained when this type is granulated with IBU A and B (Figure 3e,f) and reduced with IBU C (Figure 3g). 

#### 3.1.2. Compact Tensile Strength (TS)

**Figure 4:**
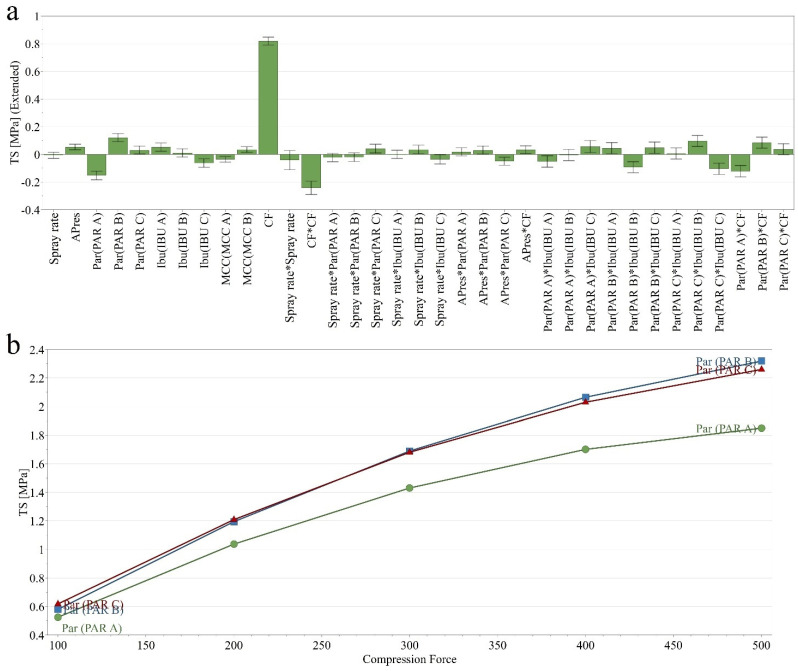
Coefficient plots representing the influence of independent variables on TS (**a**) and interaction plot between the compression force and PAR type (**b**).

The prepared granules offered differences in compactibility, reflected by the TS of the prepared compacts. The TS values ranged between 0.380 MPa and 2.735 MPa, with an Avg. ± SD of 1.500 ± 0.628 MPa (Supplementary Materials Table S2). The most relevant factor influencing this quality attribute was the compaction pressure. With increasing compaction pressure, an increase in TS was observed. Moreover, this factor exerted a non-linear effect and showed an interaction with PAR type. The applied spray rate did not influence, whereas higher atomizing pressure had a positive yet small influence on this characteristic. Considering different sources of raw materials, lower TS values were obtained when PAR A was granulated, whereas above average values were observed from PAR B (Figure 4a). 

The non-linear effect of compaction pressure and the influence of PAR type are presented in Figure 4b.

#### 3.1.3. Detachment Stress (DS) and Ejection Stress (ES)

**Figure 5:**
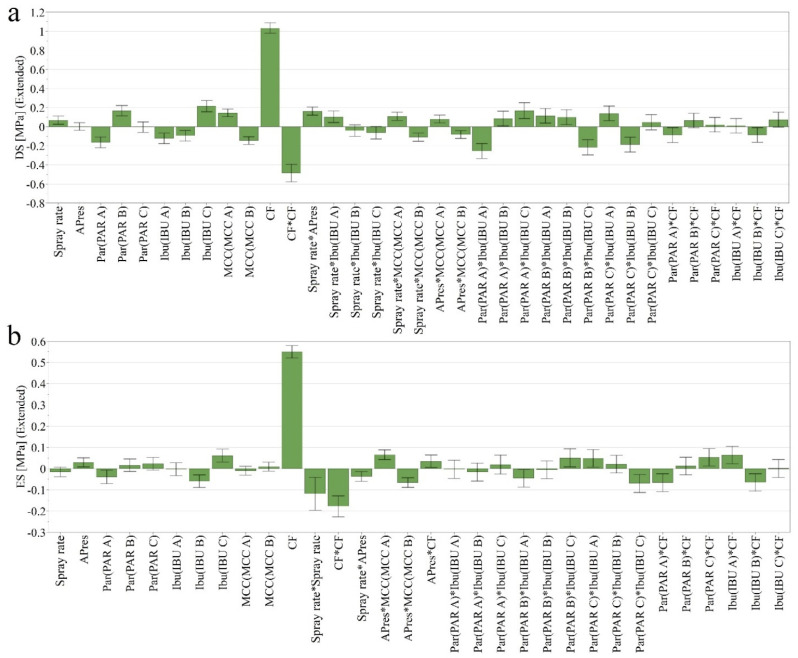
Coefficient plots representing the influence of independent variables on DS (**a**) and ES (**b**).

DS values ranged between 0.712 MPa and 4.586 MPa, with an Avg. ± SD of 2.723 ± 0.879 MPa (Supplementary Materials Table S2). Regarding the effect of compaction pressure and source of PAR on DS, the results were similar with the TS. IBU C and MCC A had a minor positive impact on the detachment profile (Figure 5a). 

ES values varied between 0.579 MPa and 2.457 MPa, presenting an Avg. ± SD of 1.423 ± 0.436 MPa (Supplementary Materials Table S2). This response was influenced mainly by compaction pressure, while a minor influence was detected from the source of IBU (Figure 5b).

#### 3.1.4. Work of Compression, Elastic Recovery and Solid Fraction

The compaction force was a significant factor for this set of responses and exerted a positive effect (figure not shown). With increasing compression force, the work of compression, the elastic recovery and the solid fraction increased. The variation range for these responses was between 624.35 J and 2216.71 J for the work of compression (Avg. ± SD: 1380.95 ± 452.72 J); 4.41% and 15.15% for elastic recovery (Avg. ± SD: 9.77 ± 3.47%), and 0.767 and 0.971 for solid fraction (Avg. ± SD: 0.884 ± 0.055) (Supplementary Materials Table S2). 

### 3.2. Quantifying the Influence of Input Variability on Compressibility, Compactibility and Tabletability Profiles

O2PLS models were developed to search for joint and unique sources of variations between X and Y data sets and to quantify the amount of correlated and orthogonal sources of variations (Table 3).

#### 3.2.1. Compressibility

The O2PLS model built to detect joint sources of variations between input factors and the variation in compressibility had the Y matrix represented by the compaction pressure and the solid fraction. The fitted model resulted that the applied compression force induced 95.6% of the variation in compaction pressure and solid fraction, as suggested by the R2Y values of the first predictive component.

**Figure 6:**
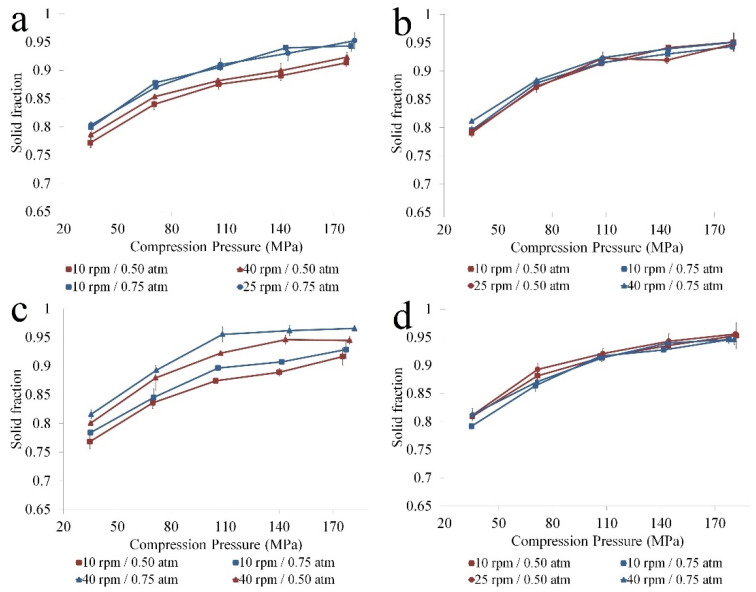
Compressibility profiles for different raw material combinations: (**a**) PAR B, IBU B; (**b**) PAR B, IBU C; (**c**) PAR A, IBU B; (**d**) PAR C, IBU B.

The second predictive component identified that approximately 9% of input variability was correlated with 4.4% variation of compact solid fraction. The coefficient plot revealed a process factor, namely the spray rate, and two raw material types (PAR B and IBU B) as being significant. The coefficient plot also showed that higher solid fractions were obtained when PAR B and IBU B were granulated using larger atomizing pressures, which was also correlated with increased span values (Supplementary Materials Figure S1). The compressibility profile generated for these raw material combinations confirmed the results of the O2PLS analysis (Figure 6). Granules prepared using PAR B and IBU B types of raw materials showed a differentiated compressibility profile with respect to the applied atomizing pressure. Higher atomizing pressures improved compressibility through the entire compaction pressure range as the obtained solid fraction values were larger (Figure 6a).

The raw material combination comprised of PAR A and IBU B also presented variability in compressibility. In this case, a grouping can be observed with respect to the applied process conditions. For this raw material combination, the compressibility improved as the atomizing pressure and the applied spray rates were increased (Figure 6c). 

In contrast, when using PAR B and IBU C, a robust performance was obtained with respect to compressibility profile. As can be seen, the differences in process conditions did not impact the ability of granules to densify under the applied compaction pressure (Figure 6b). Less variability was also observed for the PAR C and IBU B combination (Figure 6d).

The observed variability in compressibility can be explained by considering the initial particle size of APIs, the span of the resulting granules and the fragmentation ability of granules. For granules prepared from low particle size PAR B and IBU B (Figure 6a), the compressibility was sensitive to the applied atomizing pressure during binder solution addition. At low atomizing pressures, larger particles and lower span values contributed to a lower compressibility as the inter-particulate distance within the powder bed was larger, and the air voids were not filled by smaller particles (due to the lower span) (Table 4). 

A larger span value contributes to the compact densification process through an improved contact surface area and better particle packaging, thus upon compression, the solid fraction is increased. Moreover, it was shown that polydisperse samples rearrange more effectively during compression than monodisperse particles [35]. 

When PAR B was granulated with IBU C, more robust compressibility was obtained with respect to the varying process parameters (Figure 6b). The larger particles of IBU acted as agglomeration centers and offered larger particles with lower span values even under increased atomizing pressures (Table 4). Despite the lower span, the obtained solid fractions were not reduced, probably due to these granules’ larger fragmentation propensity, which effectively led to the formation of smaller particles that filled the voids more efficiently. It has been demonstrated that larger particles require smaller pressures to break, considering their lower resistance against compression [36,37]. 

Another factor to consider is the strength of the granules and the impact on volume reduction. It was demonstrated that starting with smaller initial particle size, the number of bridges that can develop under granulation increases, thus increasing the strength of the granules [34]. To this respect, the differences in compressibility of granules prepared from fine raw materials may also come from differences in granule strength and the influence of atomizing pressure on this characteristic. As smaller particles require more liquid for appropriate agglomeration, the way the liquid is applied (i.e., atomizing pressure) could have a larger influence compared to the case of coarse initial raw materials. 

The process parameters also influenced the compressibility of PAR A with IBU B. Again, the solid fraction of the prepared compacts increased by using larger atomizing pressures during binder addition. Granules prepared using intermediate size PAR (PAR C) and low size IBU (IBU B) also lead to reproducible compressibility, probably due to the larger polydispersity that allowed a better filling of the inter-particulate gaps in the powder bed. 

#### 3.2.2. Compactibility

The O2PLS model built for compactibility, revealed that 96.8% of variability was correlated with the applied compression force. The second predictive component highlighted the effect of raw materials on compact porosity. By changing the source of PAR and IBU, a 3.23% variation in compactibility was obtained. 

**Figure 7:**
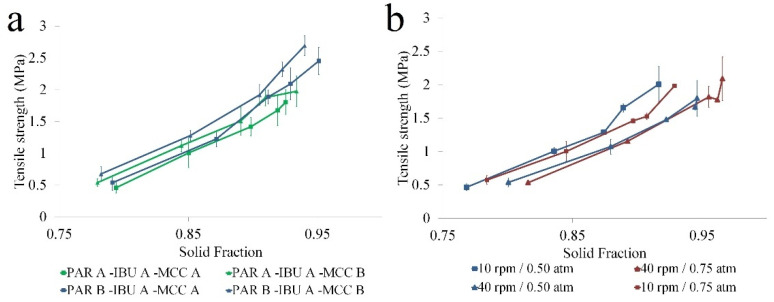
Compactibility profiles for different raw material combinations: (**a**) PAR A-B, IBU A; (**b**) PAR A, IBU B.

The coefficient plot identified significant effects for A and B types of PAR and IBU A (Supplementary Materials Figure S2). PAR A offered slightly lower TS values at the lowest compression force than PAR B under similar solid fractions (Figure 7a). The lower solid fraction obtained from PAR A-based granules at larger forces contributed to the lower TS. These results suggest that the selection of PAR source could impact the strength of the bonds, especially at lower forces (lower TS for similar solid fractions), whereas a larger impact on the inter-particulate bonding area at increased pressures. The effective bonding area increased with increasing solid fractions and contributed to compact TS. The effect between solid fraction and TS was also confirmed by Wünsch et al. [37]. 

The compactibility plot for the PAR A and the IBU B combination showed that the applied spray rate offered variability in compactibility of the granules (Figure 7b). For this PAR/IBU combination, despite the larger spray rate during granulation leading to an increased solid fraction, the TS was less influenced by this factor. 

#### 3.2.3. Tabletability

**Figure 8:**
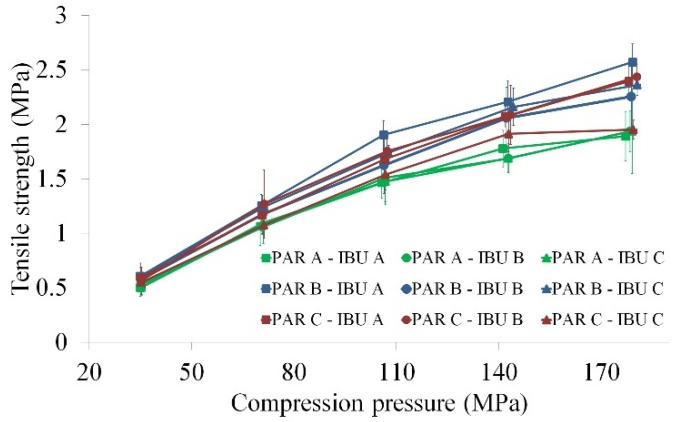
Tabletability profiles for different PAR–IBU supplier combinations (average profiles).

O2PLS analysis revealed that 96.4% variability in tabletability was well correlated with the change in compaction pressure, while the remaining 3.6% could be attributed to process- and raw material-related factors. Higher TS compacts were obtained at higher atomizing pressures, with PAR B and formulations with larger span values (Supplementary Materials Figure S3). On the contrary, when PAR A was used, the TS of the compacts was lower (Figure 8).

The TS of a compact is influenced by multiple factors, considering the inter-particulate bonding area, bond strength, particle deformation, particle fragmentation and elastic recovery [38,39,40]. Larger bonding areas consecutive of particle deformation and fragmentation are beneficial for increased mechanical resistance [38]. To this respect, the effect of the applied atomizing pressure during binder addition and the granule span was detailed when discussing the granule compressibility. Larger levels of these variables lead to improved compressibility, yielding an enhanced inter-particulate surface contact area and a consecutive higher solid fraction. It was also shown how the lower solid fractions obtained from PAR A-based granules negatively influenced compact TS.

**Figure 9:**
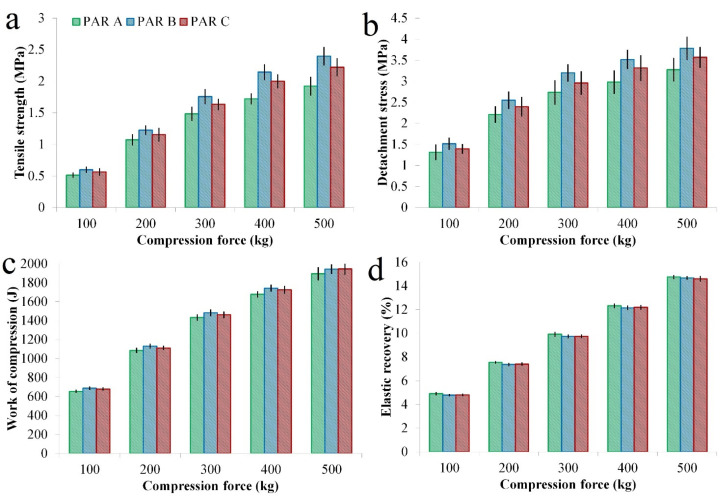
Average parameters calculated for PAR A-B-C-based granules resulted upon dynamic compaction analysis: (**a**) TS; (**b**) DS; (**c**) Work of compression; (**d**) Elastic recovery.

Comparing the average work of compression of formulations in function of PAR type, it was highlighted that the work of compression increased as the compaction pressure increased and that PAR A had lower values compared to the other types (Figure 9c). As the work of compression includes the work associated with particle rearrangement, deformation and fragmentation [41], it is evident that the lower tendency of PAR A-based granules towards these processes contributed to the lower TS. During the more pronounced fragmentation of PAR B- and PAR C-based granules, the volume reduction under particle rearrangement was increased, and the resulting fine particles enhanced the strength of the compacts [42,43]. This effect could also be held responsible for the slightly higher DS values due to increased contact points with the surface of the punch (Figure 9b).

Another aspect to consider is the elastic recovery, as during decompression, it leads to a reduction in the bonding area and TS (Figure 9d) [38,39]. Slightly larger elastic recoveries were identified for PAR A-based granules, although the obtained differences were not statistically significant. 

The moisture level is another factor that can impact the deformation behavior of the particles by influencing the elasticity and plasticity factors [44]. In this case, larger loss on drying values were identified for PAR A-based granules compared to the other types (PAR A = 4.275 ± 1.8%; PAR B = 3.750 ± 1.7%; PAR C = 3.100 ± 1.6%). Lower amounts of residual moisture reduces the elastic recovery by contributing to the formation of hydrogen bonds between particles, whereas a high water content (especially bulk water) can reduce inter-particulate interactions [40,44].

**Figure 10:**
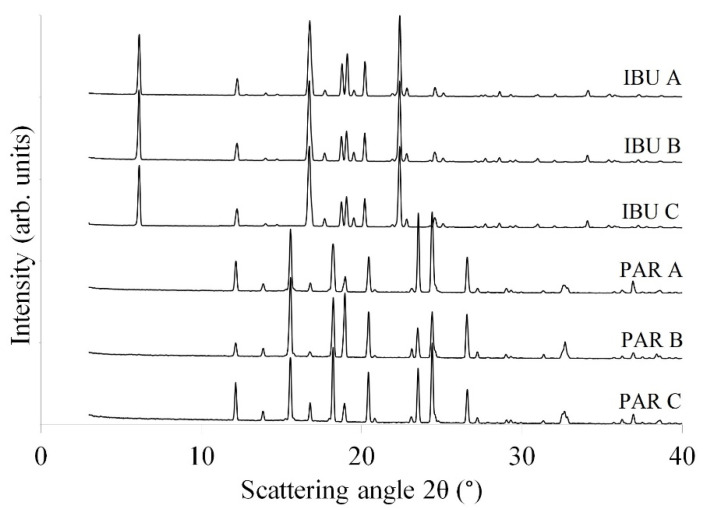
XRPD diffractograms obtained for the PAR- and IBU-type raw materials.

As no differences were detected in the crystalline structure of the used raw materials, it was concluded that the lower tabletability of PAR A-based granules is a summed contribution of the lower particle rearrangement and fragmentation, slightly higher elastic recovery and larger moisture content (Figure 10).

### 3.3. Influence of Input Variability on Compression Parameters

The calculated Py values ranged between 79.600 and 153.307 (Avg. ± SD: 118.367 ± 17.267), thus, except for one formulation that was classified as soft (Py ∈ (40, 80)), all the formulations were considered to be moderately hard (Py ∈ (80, 200)) (Supplementary Materials Table S3). The slope of the Heckel equation estimates the level of plasticity in the characterized material, with higher slope values (k_H_)/lower Py suggesting easier and more rapid plastic deformation [45]. Plastic deformation allows the development of sufficient contact area between particles, thus offering good compactibility [16]. 

Having moderately hard granules with more difficult to deform particles highlights the importance of fragmentation and the presence of fines for this particular formulation. To highlight the effect of input factors, the Py parameter was used as a response in the DoE. The obtained *p*-values suggested no significant influence from raw material- or process-related factors (Supplementary Materials Table S4). Additionally, no significant model could be fitted. 

**Figure 11:**
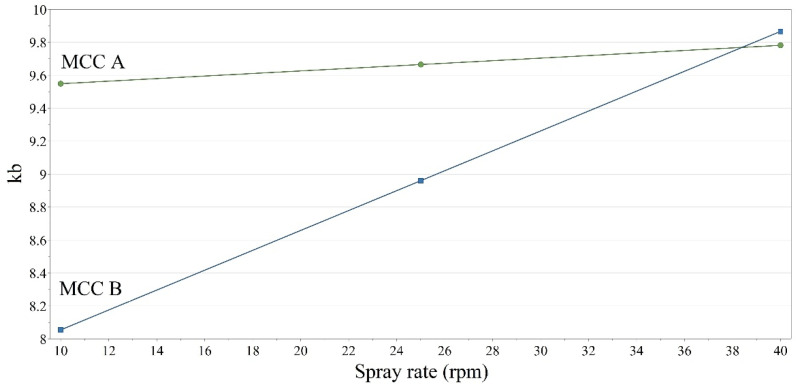
Interaction plot between the MCC type and the applied spray rate.

In case of compactibility, the k_b_ values ranged between 6.764 and 12.209 (Avg. ± SD: 9.373 ± 1.141), with most of the formulations being easily compacted (k_b_ < 10) and 9 formulations being difficultly compacted (k_b_ > 10). ANOVA test revealed a significant effect from the source of MCC (*p* = 2.05 × 10^−2^), whereas none of the other factors were identified as significant. The statistically significant effect showed a superior bonding capability for granules prepared using MCC B. Additionally, a significant interaction term was identified between this factor and the spray rate (*p* = 3.75 × 10^−2^). The interaction term revealed that the applied spray rate did not influence the kb parameter for MCC A-based granules. In contrast, for MCC B, the k_b_ value decreased (compactibility increased) as the spray rate was reduced (Figure 11). The design space generation procedure (presented under Section 3.4.) demonstrates that this statistically significant difference is also practically relevant, as the robustness of formulations depended on the diluent supplier and the applied compression force.

The parameters derived from the Power model (tabletability) ranged between 5.8 × 10^−3^−5.75 × 10^−2^ (Avg. ± SD: 3.03 × 10^−2^ ± 1.23 × 10^−2^) for d and between 0.714−1.182 (Avg. ± SD: 0.858 ± 0.098) for g. The obtained pressure sensitivity descriptor (g) values accounted for a diverse range of function shapes, having 1 concave (g > 1.05), 4 linear (0.95 > g < 1.05) and convex shapes (g < 0.95) for the remaining formulations. According to the classification system proposed by S. Dai et al., the tableting behavior was in the second category, having the d values between 2 × 10^−3^ ≤ d < 0.5 [30]. Within this interval, eight formulations presented unacceptable tabletability over the investigated pressure range, whereas the remaining formulations had acceptable tabletability (TS > 2) at middle to high compression pressures (140–180 MPa). Out of the eight formulations with unacceptable tabletability, seven corresponded to PAR A-based formulations. 

The filler supplier influenced both d and g parameters (*p* = 3.39 × 10^−3^; *p* = 1.32 × 10^−2^), with MCC A-type granules presenting lower than average tabletability capacity and above average pressure sensitivity index. For MCC B, an inverse combination was obtained.

Being processed through a high number of stages, MCC is considered an important source of batch-to-batch variability, especially for granulation processes, as concluded by multiple studies [6,46]. Comparing the material properties of the two MCC types, differences were highlighted in bulk density, tapped density, loss on drying and water binding capacity (Table 5).

The influence of bulk and tapped density on the tableting properties of MCC seems to be formulation- and application-dependent. Fang et al. stated that these parameters did not significantly affect TS, Kawakita plot and force-displacement curves. In contrast, Thoorens et al. showed an inverse correlation between tapped density and tabletability [47,48]. As these studies referred to direct compression, applying these conclusions to our product prepared by granulation is somewhat limited.

The inverse correlation identified between bulk density and moisture content of the MCC types was attributed to the inter-particulate friction, which eventually led to lower initial density when the moisture content was increased [46]. The tabletability was described to increase with an increasing moisture content of MCC [48]. However, in our case, no statistically significant difference could be highlighted in the loss on drying of the prepared granules despite the different water content of the starting material. Additionally, the differences in compactibility could not be linked to differences in granule particle size as no statistical significance was identified between classes defined by the type of MCC.

Regarding the effect of spray rate on the compactibility of the granules, a different behavior was identified between the two MCC types. Thus, we hypothesized that at lower binder addition rates, the densification of the granules was lower for the MCC B type. This affirmation is supported by MCC B’s higher water binding capacity, as during granule formation, a larger amount of water is absorbed/retained by the diluent, thus reducing the available water for granule consolidation. This effect occurred at low addition rates. At high addition rates, the densification of the granules was not influenced by the differences in water binding capacity, as there was a larger amount of water available for granule consolidation. The relationship between water binding capacity and granule consolidation was previously demonstrated by Portier et al. in a twin-screw granulation setup [46].

In the case of direct compression, the use of low bulk density MCC is better suited to counteract the poor tableting properties of APIs due to the higher dilution potential and higher roughness of particles [49]. The granulation of MCC was described to reduce its tabletability due to the densification that occurs during processing [49]. The loss of compactibility was explained by the implication of the compaction energy in tablet formation. For dense granules, a larger part of the compression force is used to break up the granules and therefore, the strength of the inter-particulate bonds are reduced [50]. The current study highlighted differences in compact TS between MCC A- and B-based granules at lower compression forces. As the compression forces increased, the differences in TS were reduced. In this case, the larger pressure sensitivity index of MCC A-based granules meant that the particles were more easily compressed into a tablet at higher pressures. In contrast, the larger tabletability capacity for MCC B provided improved compact TS even at lower pressures.

**Figure 12:**
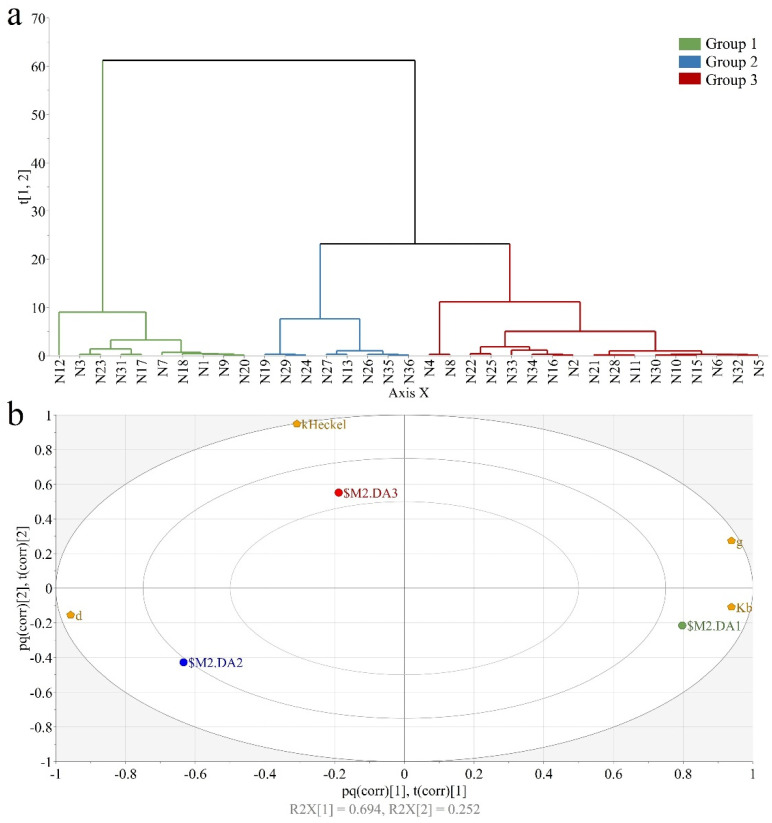
Discriminating formulations based on compression parameters: (**a**) Hierarchical cluster analysis; (**b**) Biplot of OPLS-DA model.

A PCA model was fitted on the data to evaluate how the calculated compression parameters combined to offer the overall performance of the formulations during compression. The two principal components, summarizing 94.6% of parameter variability, were used as input for Hierarchical Cluster Analysis (HCA), enabling the separation of observations into classes. The groups identified through HCA (Figure 12a) were used to define classes for the discriminant analysis. The OPLS-DA model with two significant predictive components (R2X1-0.694; R2X2-0.252) offered a good separation of observations with respect to class membership, as shown by the increased total variation (R2Ycum = 0.99) in Y matrix (dummy variable matrix, coding class membership) and the predictive capacity of (Q2 = 0.498).

By the positioning dummy variables in relation to the compression parameters, the biplot reveals how these parameters combine for classes of observations. The plot presented in Figure 12b showed that the formulations from the first class ($M2.DA1) were characterized by a larger k_b_ value (more difficultly compactable), smaller d value (lower tabletability capacity) and larger g (larger pressure sensitivity index), whereas the second class ($M2.DA2) had opposite properties. Observations found in the third class ($M2.DA3) were situated between the other two classes with respect to these parameters. Still, they presented an above average k_H_ value, suggesting a lower yield pressure (faster plastic deformation). Class membership of formulations was well correlated with the previously presented individual interpretation of compression parameters. Granules from class 1, that had poor compactibility and tabletability with respect to other formulations, have been prepared using MCC A or MCC B with large binder addition rates. Except for one, the second class members have been granulated using MCC B-type filler at low or intermediate spray rates. Regarding the third class, 13 out of 17 formulations have been prepared using B and C types of PAR, as these granules have been shown to present an improved deformation through the larger work of compression and solid fractions. 

### 3.4. Selecting a Robust Formulation—Building the Design Space

The obtained results revealed the complexity of interactions between the process variables and raw material suppliers, highlighting the importance of dynamic compaction analysis when large dose products are developed by granulation. These studies can efficiently guide the science-based selection of supplier type and the possibility to adapt the granulation process parameters in function of the used raw materials. 

As presented in the Introduction, to have a robust and well-performing formulation that can deliver consistent quality through the entire lifecycle of the product, several quality attributes should be met and should present robustness to process-related variability. As the formulation robustness is highly dependent on the supplier, a design space approach was selected to identify the best performing raw material combinations. Formulation robustness to variable processing conditions can be easily evaluated as the size of the area in the experimental region of process factors associated with a low risk of failure. 

The design space was computed for the investigated raw material combinations considering granules’ size and tableting properties. In the optimizer of Modde Software, the following restrictions were defined: granules should present an average particle size of 300 µm ± 20 µm; a span value below 2; whereas upon compaction, the prepared compact should present a TS above 2 MPa, with DS and ES values below 3 MPa [51,52]. 

**Figure 13:**
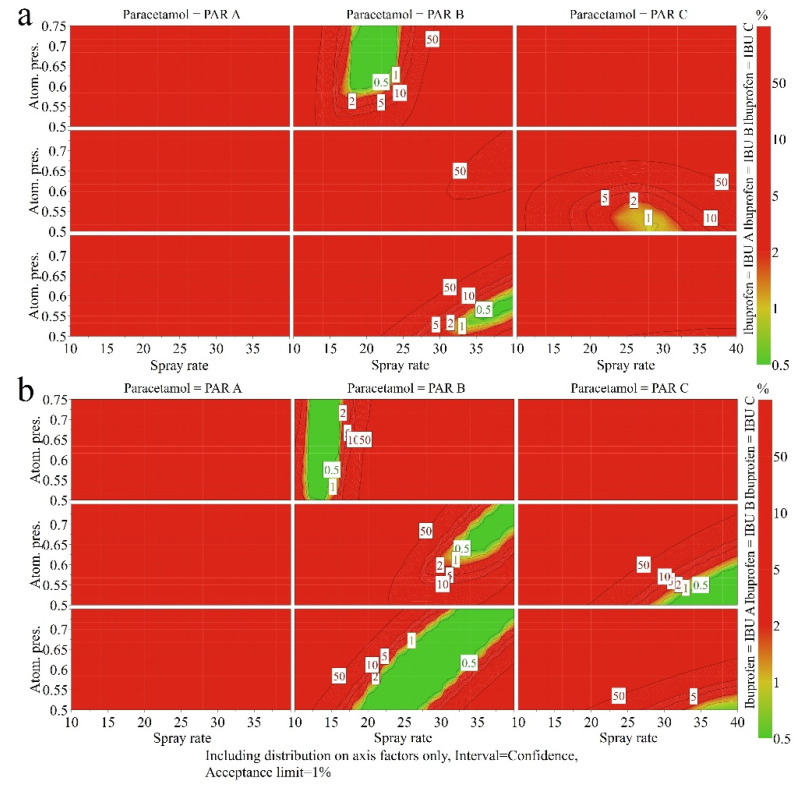
4D design space plots for the granulation process, considering a compression force of 400 kg and MCC A (**a**) versus MCC B (**b**) types of diluent.

The source of MCC was important to ensure the desired quality. Considering the allowed variation of process factors during fluid bed granulation and the available API raw material suppliers, working with a compaction pressure of 400 kg offered a more robust granule performance for MCC B (Figure 13). Granules prepared using PAR B and IBU A showed the largest tolerance in the way the binder solution can be applied, as suggested by the large area from the experimental region associated with a probability of failure below 1%. For this raw material combination (PAR B, IBU A, MCC B), the applied atomizing pressure should be increased with an increasing spraying rate. If the increased addition of binder solution is compensated by reducing the size of droplets, it is possible to obtain a product with identical performance.

When MCC B is used, PAR B can also be granulated with the other two types of IBU in different conditions and by using a more restricted variation of process parameters. For IBU B, higher spray rates are required to reach the required target particle size and higher atomizing pressures are needed for improved compressibility. When granulating in the presence of IBU C, lower spray rates are necessary to ensure a limited particle growth in the presence of large IBU particles. However, the applied atomizing pressure can vary independently as the compressibility was unaffected (see Section 3.2.1.).

On the other side, if PAR C is granulated in the presence of MCC B, only A and B types of IBU can deliver the desired properties if compacts are compressed using 400 kg load.

In the case of MCC A and a 400 kg compression force, only the PAR B–IBU A and PAR B–IBU C combinations have a low risk of failure. However, the robustness to varying process factors is poor compared to MCC B.

**Figure 14:**
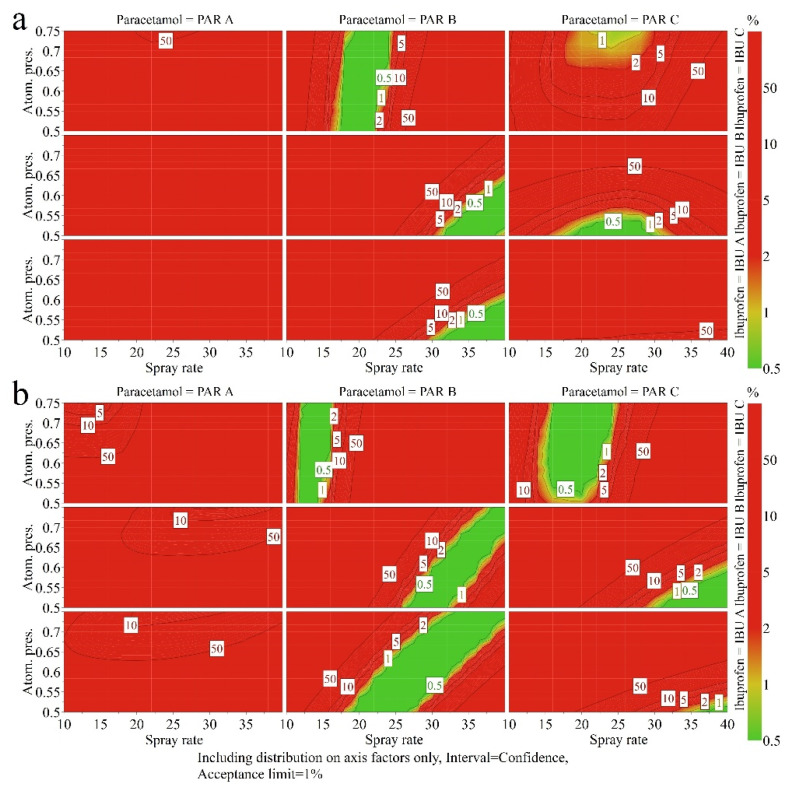
4D design space plots for the granulation process, considering a compression force of 500 kg and MCC A (**a**) versus MCC B (**b**) types of diluent.

When a 500 kg compression force is used for tablet preparation, the differences observed between the two MCC types are reduced compared to a lower compression force (Figure 14).

The obtained differences between the MCC suppliers at the two compression forces can be explained through the estimated compression parameters. MCC A-based formulations were more difficultly compactable. Additionally, they presented lower than average tabletability capacity, explaining the differences in the size of green areas (low risk of failure) in the design space at 400 kg compression. The same formulations presented a larger pressure sensitivity index, meaning that the granules were easier compressed into a tablet at higher pressures. This explains the changes in design space plots when a 500 kg compression force is applied. For MCC B-based granules, due to the improved compactibility and tabletability, appropriate tablets could be prepared at both compression forces.

Even at a larger compression force, PAR A is not favorable, as a high risk of failure was present for all the possible process/raw material combinations. In the case of PAR B–IBU C combination, the spray rate should be slightly decreased when MCC B is used instead of MCC A due to the influence on granule compactibility. In both cases, the applied atomizing pressure is not important, as long as the 0.5–0.75 atm range is respected. 

For PAR B–IBU A and PAR B–IBU B combinations, the spray rate can be varied, but the atomizing pressure should be set accordingly. For MCC A-based formulations, the binder addition rate should be placed towards the top end of the investigated region, whereas the atomizing pressure can be increased from 0.5 atm up to 0.6–0.65 atm. In the case of MCC B, the spray rate and atomizing pressure can be varied in a larger range and the formulation is more robust.

The PAR C–IBU C combination can deliver the required response profile when a 500 kg compression force is applied. The MCC B-type diluent offers an improved tolerance towards varying process factors. The PAR C–IBU B formulations should be processed using lower atomizing pressures and average spray rates for MCC A, respectively, high spray rates for MCC B.

### 3.5. Practical Implications

This study presents an experimental strategy with an important impact on the industrial development of medicines to ensure the robustness of the product and an adequate response to the sources of variability that may occur throughout its lifecycle. It describes a sequential approach in which each step supplements the understanding and ability to control the process. The implementation of such a strategy for the development of solid pharmaceutical forms should be based on an assessment of the required cost-time versus the gained information. 

In this study, the DoE phase revealed the effects of critical material characteristics and granulation process parameters on several outputs: granule size, span and compression parameters. In the next stage, MVDA enabled the inclusion of granule characteristics as input variables together with the DoE factors, and the analysis of their impact on the compressibility, compactability and tabletability profiles. The HCA enabled the grouping of materials into classes according to the compactibility and tabletability capacity. Finally, the DS development allowed the defining optimal formulations and conditions with the best performances; moreover, it enabled the use of processing parameters to correct the faults derived from material variability. According to the needed information and required degree of understanding, development scientists can select a particular stage of analysis that meets their needs.

## 4. Conclusions

The present study demonstrates the utility of dynamic compaction analysis in the formulation development of pharmaceutical granules with a complex composition. The importance of this analysis stands out from the perspective of the correct selection of raw material suppliers and the necessity to adapt processing conditions during granule preparation to ensure the same downstream processing ability.

The initial particle size of the raw materials influenced granule size and polydispersity. Larger initial particle sizes for the APIs lead to larger and more uniform granules. The type of PAR influenced product compressibility, which was related to the span and fragmentation ability of the granules. Moreover, the influence of the atomizing pressure on compressibility became significant when different PAR types were granulated in the presence of smaller particle sizes of IBU. In this case, higher atomizing pressures lead to more polydisperse granules and larger solid fractions upon compaction.

Using PAR types with larger initial particle size (PAR A) produced granules with lower tabletability due to the lower rearrangement, lower fragmentation, slightly higher elastic recovery and, possibly, to the higher residual moisture content of the granules. The improved tabletability of PAR B-based granules enabled the identification of design space at both compression forces, whereas an increased compression force was required for PAR C. These types offered a better fragmentation at higher compression pressures and produced more fines, needed for a larger effective bonding area and stronger mechanical resistance.

Changing the supplier of IBU influenced the particle size and span values, as well as presented an interaction with PAR regarding the compressibility of the granules. The variability related to this API was efficiently controlled by adapting the spray rate and atomizing pressure. In general, middle to high spray rates with increasing atomizing pressures were required for the fine types (IBU A-B) and lower binder addition rates independent of atomizing pressure for the coarser grade (IBU C). Moreover, the required binder addition conditions were also dependent on the MCC type.

By fitting mathematical models on the available compression data, the influence of MCC type on granule compactibility (ease of compaction) and tabletability (tableting capacity; pressure sensitivity index) was highlighted. Differences in water binding capacity affected granule consolidation, especially under low spray rates. In this respect, MCC B’s higher water binding capacity offered granules with better downstream processability.

Building the design space for different raw material combinations enabled the identification of the most robust formulations that tolerate larger variations in the way the binder solution is applied. The associated quality variations were minimized for these raw material combinations, thus ensuring a reproducible performance and consistent tableting properties. 

## Figures and Tables

**Table 1:** Independent variables included in the experimental design.

Factor Name	Type	Investigated Variation Levels
Spray rate (rpm)	quantitative	10 (5 g/min); 25 (12.5 g/min); 40 (20 g/min)
Atomizing pressure (atm)	quantitative	0.5; 0.75
PAR type	qualitative	PAR-A; PAR-B; PAR-C
IBU type	qualitative	IBU-A; IBU-B; IBU-C
MCC type	qualitative	MCC-A; MCC-B

**Table 2:** Model performance parameters for the DoE work-set.

Response	R2	Q2	Validity	Reproducibility	ANOVA Regression	ANOVA Lack of Fit
Xa	0.877	0.835	0.606	0.965	2.120 × 10^−5^	2.074 × 10^−1^
Span	0.835	0.776	0.936	0.685	1.823 × 10^−4^	7.746 × 10^−1^
TS	0.960	0.947	0.473	0.993	6.981 × 10^−9^	1.221 × 10^−1^
DS	0.925	0.902	0.748	0.953	1.490 × 10^−6^	3.654 × 10^−1^
ES	0.911	0.885	0.442	0.987	6.432 × 10^−6^	1.075 × 10^−1^
Work of compression	0.989	0.974	0.311	0.999	1.860 × 10^−15^	6.383 × 10^−2^
Elastic recovery	0.997	0.952	0.510	0.999	1.111 × 10^−17^	1.414 × 10^−1^
Solid fraction	0.874	0.800	0.426	0.984	9.166 × 10^−5^	1.008 × 10^−1^

Xa—average particle size; TS—tensile strength; DS—detachment stress; ES—ejection stress.

**Table 3:** O2PLS model characteristics.

	Compressibility	Compactibility	Tabletability
Modeled Responses	Compaction Pressure & Solid Fraction	TS & Solid Fraction	Compaction Pressure & TS
Model structure	2 + 2 + 0	2 + 2 + 0	2 + 2 + 0
R2X (P1)	0.063	0.067	0.066
R2X (P2)	0.089	0.108	0.120
R2X (O1)	0.265	0.272	0.265
R2X (O2)	0.142	0.138	0.139
R2Y (P1)	0.956	0.968	0.964
R2Y (P2)	0.044	0.032	0.036
Q2	0.914	0.861	0.947

P—predictive component; O—orthogonal component.

**Table 4:** Particle size and span differences of different PAR and IBU combinations.

Exp No.	Spray Rate	Atomizing Pressure	PAR	IBU	Xa	Span
N7	10	0.5	PAR A	IBU B	287	1.6
N26	10	0.75	PAR A	IBU B	257	1.5
N25	40	0.5	PAR A	IBU B	329	1.7
N8	40	0.75	PAR A	IBU B	280	1.7
N27	10	0.5	PAR B	IBU B	275	1.6
N10	10	0.75	PAR B	IBU B	215	1.8
N28	25	0.75	PAR B	IBU B	268	1.6
N9	40	0.5	PAR B	IBU B	310	1.7
N33	10	0.5	PAR B	IBU C	280	1.5
N16	10	0.75	PAR B	IBU C	287	1.4
N15	25	0.5	PAR B	IBU C	322	1.4
N34	40	0.75	PAR B	IBU C	369	1.5
N29	10	0.5	PAR C	IBU B	249	1.7
N12	10	0.75	PAR C	IBU B	294	2.1
N11	25	0.5	PAR C	IBU B	280	1.8
N30	40	0.75	PAR C	IBU B	290	1.7

**Table 5:** Physical properties of MCC types.

Material Properties	MCC A	MCC B
d50 (µm)	46.021	48.600
Bulk density (g/mL)	0.353 ± 0.004	0.340 ± 0.002
Tapped density (g/mL)	0.442 ± 0.002	0.461 ± 0.001
Loss on drying (%)	2.330 ± 0.3 × 10^−3^	4.537 ± 0.6 × 10^−3^
Water binding capacity (%)	208.209 ± 0.905	221.431 ± 2.990

## Data Availability

The data presented in this study are available upon request from the corresponding author.

## Supplementary Materials

The following supporting information can be downloaded at: https://www.mdpi.com/article/10.3390/pharmaceutics14010177/s1, Figure S1: pq loading plot for the first and second predictive component of the O2PLS model fitted for compressibility, Figure S2: pq loading plot for the first and second predictive component of the O2PLS model fitted for compactibility, Figure S3: pq loading plot for the first and second predictive component of the O2PLS model fitted for tabletability, Table S1: DoE workset—factor combination (39 runs x blocked on 5 levels of CF), Table S2: DoE workset—response matrix, Table S3: Estimated compression parameters, Table S4: Influence of raw material- and process-related factors on compression parameters.

## Abbreviations

Active pharmaceutical ingredient—API; detachment stress—DS; ejection stress—ES; ibuprofen—IBU; microcrystalline cellulose—MCC; paracetamol—PAR; scanning electron microscopy—SEM; tensile strength—TS; X-ray Powder Diffraction—XRPD.
