# Supplementary material for: In-Depth Understanding of Granule Compression Behavior under Variable Raw Material and Processing Conditions
Source: Pharmaceutics. 2022 Jan 12;14(1):177. doi: 10.3390/pharmaceutics14010177 (PMC8780340; doi:10.3390/pharmaceutics14010177)
Supplement: Supplementary file 1 [file pharmaceutics-14-00177-s001.zip › pharmaceutics-1530635-supplementary.pdf]

pharmaceutics

MDPI

Supplementary Materials: In-Depth Understanding of Granule Compression Behavior under Variable Raw Material and Processing Conditions

Tibor Casian, Sonia Iurian, Alexandru Gâvan, Alina Porfire, Anca Lucia Pop, Simona Crișan, Anda Maria Pușcaș and Ioan Tomuță

Table S1. DoE workset – factor combination (39 runs x blocked on 5 levels of CF).

Exp. No.	Exp. Name	Spray Rate	Atomizing Pressure	API 1 - sort	API 2 - sort	Filler - Sort	CF
1	N1_Block_1	40	0.5	PAR A	IBU A	MCC A	100
2	N2_Block_1	10	0.75	PAR A	IBU A	MCC A	100
3	N3_Block_1	10	0.5	PAR B	IBU A	MCC A	100
4	N4_Block_1	40	0.75	PAR B	IBU A	MCC A	100
5	N5_Block_1	10	0.5	PAR C	IBU A	MCC A	100
6	N6_Block_1	40	0.75	PAR C	IBU A	MCC A	100
7	N7_Block_1	10	0.5	PAR A	IBU B	MCC A	100
8	N8_Block_1	40	0.75	PAR A	IBU B	MCC A	100
9	N9_Block_1	40	0.5	PAR B	IBU B	MCC A	100
10	N10_Block_1	10	0.75	PAR B	IBU B	MCC A	100
11	N11_Block_1	25	0.5	PAR C	IBU B	MCC A	100
12	N12_Block_1	10	0.75	PAR C	IBU B	MCC A	100
13	N13_Block_1	10	0.5	PAR A	IBU C	MCC A	100
14	N14_Block_1	40	0.75	PAR A	IBU C	MCC A	100
15	N15_Block_1	25	0.5	PAR B	IBU C	MCC A	100
16	N16_Block_1	10	0.75	PAR B	IBU C	MCC A	100
17	N17_Block_1	40	0.5	PAR C	IBU C	MCC A	100
18	N18_Block_1	10	0.75	PAR C	IBU C	MCC A	100
19	N19_Block_1	10	0.5	PAR A	IBU A	MCC B	100
20	N20_Block_1	40	0.75	PAR A	IBU A	MCC B	100
21	N21_Block_1	40	0.5	PAR B	IBU A	MCC B	100
22	N22_Block_1	10	0.75	PAR B	IBU A	MCC B	100
23	N23_Block_1	40	0.5	PAR C	IBU A	MCC B	100
24	N24_Block_1	10	0.75	PAR C	IBU A	MCC B	100
25	N25_Block_1	40	0.5	PAR A	IBU B	MCC B	100
26	N26_Block_1	10	0.75	PAR A	IBU B	MCC B	100
27	N27_Block_1	10	0.5	PAR B	IBU B	MCC B	100
28	N28_Block_1	25	0.75	PAR B	IBU B	MCC B	100
29	N29_Block_1	10	0.5	PAR C	IBU B	MCC B	100
30	N30_Block_1	40	0.75	PAR C	IBU B	MCC B	100
31	N31_Block_1	40	0.5	PAR A	IBU C	MCC B	100
32	N32_Block_1	10	0.75	PAR A	IBU C	MCC B	100
33	N33_Block_1	10	0.5	PAR B	IBU C	MCC B	100
34	N34_Block_1	40	0.75	PAR B	IBU C	MCC B	100
35	N35_Block_1	10	0.5	PAR C	IBU C	MCC B	100
36	N36_Block_1	25	0.75	PAR C	IBU C	MCC B	100

37	N37_Block_1	25	0.75	PAR C	IBU C	MCC B	100
38	N38_Block_1	25	0.75	PAR C	IBU C	MCC B	100
39	N39_Block_1	25	0.75	PAR C	IBU C	MCC B	100
40	N1_Block_2	40	0.5	PAR A	IBU A	MCC A	200
41	N2_Block_2	10	0.75	PAR A	IBU A	MCC A	200
42	N3_Block_2	10	0.5	PAR B	IBU A	MCC A	200
43	N4_Block_2	40	0.75	PAR B	IBU A	MCC A	200
44	N5_Block_2	10	0.5	PAR C	IBU A	MCC A	200
45	N6_Block_2	40	0.75	PAR C	IBU A	MCC A	200
46	N7_Block_2	10	0.5	PAR A	IBU B	MCC A	200
47	N8_Block_2	40	0.75	PAR A	IBU B	MCC A	200
48	N9_Block_2	40	0.5	PAR B	IBU B	MCC A	200
49	N10_Block_2	10	0.75	PAR B	IBU B	MCC A	200
50	N11_Block_2	25	0.5	PAR C	IBU B	MCC A	200
51	N12_Block_2	10	0.75	PAR C	IBU B	MCC A	200
52	N13_Block_2	10	0.5	PAR A	IBU C	MCC A	200
53	N14_Block_2	40	0.75	PAR A	IBU C	MCC A	200
54	N15_Block_2	25	0.5	PAR B	IBU C	MCC A	200
55	N16_Block_2	10	0.75	PAR B	IBU C	MCC A	200
56	N17_Block_2	40	0.5	PAR C	IBU C	MCC A	200
57	N18_Block_2	10	0.75	PAR C	IBU C	MCC A	200
58	N19_Block_2	10	0.5	PAR A	IBU A	MCC B	200
59	N20_Block_2	40	0.75	PAR A	IBU A	MCC B	200
60	N21_Block_2	40	0.5	PAR B	IBU A	MCC B	200
61	N22_Block_2	10	0.75	PAR B	IBU A	MCC B	200
62	N23_Block_2	40	0.5	PAR C	IBU A	MCC B	200
63	N24_Block_2	10	0.75	PAR C	IBU A	MCC B	200
64	N25_Block_2	40	0.5	PAR A	IBU B	MCC B	200
65	N26_Block_2	10	0.75	PAR A	IBU B	MCC B	200
66	N27_Block_2	10	0.5	PAR B	IBU B	MCC B	200
67	N28_Block_2	25	0.75	PAR B	IBU B	MCC B	200
68	N29_Block_2	10	0.5	PAR C	IBU B	MCC B	200
69	N30_Block_2	40	0.75	PAR C	IBU B	MCC B	200
70	N31_Block_2	40	0.5	PAR A	IBU C	MCC B	200
71	N32_Block_2	10	0.75	PAR A	IBU C	MCC B	200
72	N33_Block_2	10	0.5	PAR B	IBU C	MCC B	200
73	N34_Block_2	40	0.75	PAR B	IBU C	MCC B	200
74	N35_Block_2	10	0.5	PAR C	IBU C	MCC B	200
75	N36_Block_2	25	0.75	PAR C	IBU C	MCC B	200
76	N37_Block_2	25	0.75	PAR C	IBU C	MCC B	200
77	N38_Block_2	25	0.75	PAR C	IBU C	MCC B	200
78	N39_Block_2	25	0.75	PAR C	IBU C	MCC B	200
79	N1_Block_3	40	0.5	PAR A	IBU A	MCC A	300
80	N2_Block_3	10	0.75	PAR A	IBU A	MCC A	300
81	N3_Block_3	10	0.5	PAR B	IBU A	MCC A	300
82	N4_Block_3	40	0.75	PAR B	IBU A	MCC A	300
83	N5_Block_3	10	0.5	PAR C	IBU A	MCC A	300
84	N6_Block_3	40	0.75	PAR C	IBU A	MCC A	300
85	N7_Block_3	10	0.5	PAR A	IBU B	MCC A	300
86	N8_Block_3	40	0.75	PAR A	IBU B	MCC A	300

87	N9_Block_3	40	0.5	PAR B	IBU B	MCC A	300
88	N10_Block_3	10	0.75	PAR B	IBU B	MCC A	300
89	N11_Block_3	25	0.5	PAR C	IBU B	MCC A	300
90	N12_Block_3	10	0.75	PAR C	IBU B	MCC A	300
91	N13_Block_3	10	0.5	PAR A	IBU C	MCC A	300
92	N14_Block_3	40	0.75	PAR A	IBU C	MCC A	300
93	N15_Block_3	25	0.5	PAR B	IBU C	MCC A	300
94	N16_Block_3	10	0.75	PAR B	IBU C	MCC A	300
95	N17_Block_3	40	0.5	PAR C	IBU C	MCC A	300
96	N18_Block_3	10	0.75	PAR C	IBU C	MCC A	300
97	N19_Block_3	10	0.5	PAR A	IBU A	MCC B	300
98	N20_Block_3	40	0.75	PAR A	IBU A	MCC B	300
99	N21_Block_3	40	0.5	PAR B	IBU A	MCC B	300
100	N22_Block_3	10	0.75	PAR B	IBU A	MCC B	300
101	N23_Block_3	40	0.5	PAR C	IBU A	MCC B	300
102	N24_Block_3	10	0.75	PAR C	IBU A	MCC B	300
103	N25_Block_3	40	0.5	PAR A	IBU B	MCC B	300
104	N26_Block_3	10	0.75	PAR A	IBU B	MCC B	300
105	N27_Block_3	10	0.5	PAR B	IBU B	MCC B	300
106	N28_Block_3	25	0.75	PAR B	IBU B	MCC B	300
107	N29_Block_3	10	0.5	PAR C	IBU B	MCC B	300
108	N30_Block_3	40	0.75	PAR C	IBU B	MCC B	300
109	N31_Block_3	40	0.5	PAR A	IBU C	MCC B	300
110	N32_Block_3	10	0.75	PAR A	IBU C	MCC B	300
111	N33_Block_3	10	0.5	PAR B	IBU C	MCC B	300
112	N34_Block_3	40	0.75	PAR B	IBU C	MCC B	300
113	N35_Block_3	10	0.5	PAR C	IBU C	MCC B	300
114	N36_Block_3	25	0.75	PAR C	IBU C	MCC B	300
115	N37_Block_3	25	0.75	PAR C	IBU C	MCC B	300
116	N38_Block_3	25	0.75	PAR C	IBU C	MCC B	300
117	N39_Block_3	25	0.75	PAR C	IBU C	MCC B	300
118	N1_Block_4	40	0.5	PAR A	IBU A	MCC A	400
119	N2_Block_4	10	0.75	PAR A	IBU A	MCC A	400
120	N3_Block_4	10	0.5	PAR B	IBU A	MCC A	400
121	N4_Block_4	40	0.75	PAR B	IBU A	MCC A	400
122	N5_Block_4	10	0.5	PAR C	IBU A	MCC A	400
123	N6_Block_4	40	0.75	PAR C	IBU A	MCC A	400
124	N7_Block_4	10	0.5	PAR A	IBU B	MCC A	400
125	N8_Block_4	40	0.75	PAR A	IBU B	MCC A	400
126	N9_Block_4	40	0.5	PAR B	IBU B	MCC A	400
127	N10_Block_4	10	0.75	PAR B	IBU B	MCC A	400
128	N11_Block_4	25	0.5	PAR C	IBU B	MCC A	400
129	N12_Block_4	10	0.75	PAR C	IBU B	MCC A	400
130	N13_Block_4	10	0.5	PAR A	IBU C	MCC A	400
131	N14_Block_4	40	0.75	PAR A	IBU C	MCC A	400
132	N15_Block_4	25	0.5	PAR B	IBU C	MCC A	400
133	N16_Block_4	10	0.75	PAR B	IBU C	MCC A	400
134	N17_Block_4	40	0.5	PAR C	IBU C	MCC A	400
135	N18_Block_4	10	0.75	PAR C	IBU C	MCC A	400
136	N19_Block_4	10	0.5	PAR A	IBU A	MCC B	400

137	N20_Block_4	40	0.75	PAR A	IBU A	MCC B	400
138	N21_Block_4	40	0.5	PAR B	IBU A	MCC B	400
139	N22_Block_4	10	0.75	PAR B	IBU A	MCC B	400
140	N23_Block_4	40	0.5	PAR C	IBU A	MCC B	400
141	N24_Block_4	10	0.75	PAR C	IBU A	MCC B	400
142	N25_Block_4	40	0.5	PAR A	IBU B	MCC B	400
143	N26_Block_4	10	0.75	PAR A	IBU B	MCC B	400
144	N27_Block_4	10	0.5	PAR B	IBU B	MCC B	400
145	N28_Block_4	25	0.75	PAR B	IBU B	MCC B	400
146	N29_Block_4	10	0.5	PAR C	IBU B	MCC B	400
147	N30_Block_4	40	0.75	PAR C	IBU B	MCC B	400
148	N31_Block_4	40	0.5	PAR A	IBU C	MCC B	400
149	N32_Block_4	10	0.75	PAR A	IBU C	MCC B	400
150	N33_Block_4	10	0.5	PAR B	IBU C	MCC B	400
151	N34_Block_4	40	0.75	PAR B	IBU C	MCC B	400
152	N35_Block_4	10	0.5	PAR C	IBU C	MCC B	400
153	N36_Block_4	25	0.75	PAR C	IBU C	MCC B	400
154	N37_Block_4	25	0.75	PAR C	IBU C	MCC B	400
155	N38_Block_4	25	0.75	PAR C	IBU C	MCC B	400
156	N39_Block_4	25	0.75	PAR C	IBU C	MCC B	400
157	N1_Block_5	40	0.5	PAR A	IBU A	MCC A	500
158	N2_Block_5	10	0.75	PAR A	IBU A	MCC A	500
159	N3_Block_5	10	0.5	PAR B	IBU A	MCC A	500
160	N4_Block_5	40	0.75	PAR B	IBU A	MCC A	500
161	N5_Block_5	10	0.5	PAR C	IBU A	MCC A	500
162	N6_Block_5	40	0.75	PAR C	IBU A	MCC A	500
163	N7_Block_5	10	0.5	PAR A	IBU B	MCC A	500
164	N8_Block_5	40	0.75	PAR A	IBU B	MCC A	500
165	N9_Block_5	40	0.5	PAR B	IBU B	MCC A	500
166	N10_Block_5	10	0.75	PAR B	IBU B	MCC A	500
167	N11_Block_5	25	0.5	PAR C	IBU B	MCC A	500
168	N12_Block_5	10	0.75	PAR C	IBU B	MCC A	500
169	N13_Block_5	10	0.5	PAR A	IBU C	MCC A	500
170	N14_Block_5	40	0.75	PAR A	IBU C	MCC A	500
171	N15_Block_5	25	0.5	PAR B	IBU C	MCC A	500
172	N16_Block_5	10	0.75	PAR B	IBU C	MCC A	500
173	N17_Block_5	40	0.5	PAR C	IBU C	MCC A	500
174	N18_Block_5	10	0.75	PAR C	IBU C	MCC A	500
175	N19_Block_5	10	0.5	PAR A	IBU A	MCC B	500
176	N20_Block_5	40	0.75	PAR A	IBU A	MCC B	500
177	N21_Block_5	40	0.5	PAR B	IBU A	MCC B	500
178	N22_Block_5	10	0.75	PAR B	IBU A	MCC B	500
179	N23_Block_5	40	0.5	PAR C	IBU A	MCC B	500
180	N24_Block_5	10	0.75	PAR C	IBU A	MCC B	500
181	N25_Block_5	40	0.5	PAR A	IBU B	MCC B	500
182	N26_Block_5	10	0.75	PAR A	IBU B	MCC B	500
183	N27_Block_5	10	0.5	PAR B	IBU B	MCC B	500
184	N28_Block_5	25	0.75	PAR B	IBU B	MCC B	500
185	N29_Block_5	10	0.5	PAR C	IBU B	MCC B	500
186	N30_Block_5	40	0.75	PAR C	IBU B	MCC B	500

187	N31_Block_5	40	0.5	PAR A	IBU C	MCC B	500
188	N32_Block_5	10	0.75	PAR A	IBU C	MCC B	500
189	N33_Block_5	10	0.5	PAR B	IBU C	MCC B	500
190	N34_Block_5	40	0.75	PAR B	IBU C	MCC B	500
191	N35_Block_5	10	0.5	PAR C	IBU C	MCC B	500
192	N36_Block_5	25	0.75	PAR C	IBU C	MCC B	500
193	N37_Block_5	25	0.75	PAR C	IBU C	MCC B	500
194	N38_Block_5	25	0.75	PAR C	IBU C	MCC B	500
195	N39_Block_5	25	0.75	PAR C	IBU C	MCC B	500

Table S2. DoE workset – response matrix.

Exp. No.	Exp Name	Xa	span	TS	DS	ES	WoC	ER	SF
1	N1_Block_1	299	1.5	0.396	1.200	0.628	636.861	5.220	0.785
2	N2_Block_1	253	1.4	0.528	0.712	0.634	654.037	5.026	0.804
3	N3_Block_1	247	1.5	0.451	1.587	0.649	705.085	4.562	0.779
4	N4_Block_1	291	1.6	0.625	1.990	0.698	696.165	4.879	0.803
5	N5_Block_1	299	1.5	0.587	1.337	0.649	722.223	4.742	0.767
6	N6_Block_1	237	2.1	0.616	1.792	0.770	680.367	4.853	0.814
7	N7_Block_1	287	1.6	0.465	1.165	0.816	689.054	4.963	0.768
8	N8_Block_1	280	1.7	0.536	1.873	0.701	647.921	4.803	0.816
9	N9_Block_1	310	1.7	0.487	1.502	0.579	631.652	4.803	0.786
10	N10_Block_1	215	1.8	0.617	1.777	0.927	719.873	4.833	0.799
11	N11_Block_1	280	1.8	0.588	1.601	0.666	672.380	4.833	0.811
12	N12_Block_1	294	2.1	0.381	1.170	0.877	642.127	4.786	0.792
13	N13_Block_1	254	1.5	0.536	1.605	0.840	639.978	4.712	0.795
14	N14_Block_1	350	1.4						
15	N15_Block_1	322	1.4	0.566	1.551	0.866	687.194	4.695	0.793
16	N16_Block_1	287	1.4	0.602	1.619	0.862	668.282	4.783	0.796
17	N17_Block_1	338	1.7	0.427	1.292	0.709	624.353	4.916	0.769
18	N18_Block_1	265	1.2	0.447	1.528	0.677	653.880	4.777	0.778
19	N19_Block_1	295	1.4	0.577	1.514	0.906	681.756	4.742	0.775
20	N20_Block_1	311	1.7	0.502	1.166	0.645	659.089	4.978	0.785
21	N21_Block_1	326	1.6	0.612	1.214	0.618	681.315	4.969	0.782
22	N22_Block_1	266	1.7	0.740	1.245	0.741	700.142	4.776	0.783
23	N23_Block_1	301	1.8	0.463	1.263	0.866	707.659	4.753	0.775
24	N24_Block_1	245	1.5	0.701	1.175	0.695	695.786	5.018	0.788
25	N25_Block_1	329	1.7	0.539	1.179	0.757	637.645	4.820	0.801
26	N26_Block_1	257	1.5	0.572	1.283	0.772	663.960	5.048	0.784
27	N27_Block_1	275	1.6	0.569	1.558	0.736	664.849	4.902	0.772
28	N28_Block_1	268	1.6	0.627	1.233	0.845	688.045	4.768	0.803
29	N29_Block_1	249	1.7	0.660	1.252	0.722	692.874	4.850	0.810
30	N30_Block_1	290	1.7	0.634	1.438	0.653	692.326	4.834	0.813
31	N31_Block_1	387	1.5	0.423	1.418	1.171	641.473	4.806	0.797
32	N32_Block_1	299	1.3	0.557	1.314	0.698	662.512	4.829	0.805
33	N33_Block_1	280	1.5	0.637	1.510	0.669	714.812	4.792	0.790
34	N34_Block_1	369	1.5	0.631	1.385	0.975	708.752	4.665	0.811
35	N35_Block_1	290	1.4	0.563	1.701	0.825	699.322	4.408	0.774
36	N36_Block_1	312	1.5	0.607	1.325	1.045	675.029	4.772	0.807
37	N37_Block_1	312	1.4	0.607	1.325	1.045	675.029	4.772	0.807
38	N38_Block_1	335	1.7	0.607	1.325	1.045	675.029	4.772	0.807

39	N39_Block_1	324	1.6	0.607	1.325	1.045	675.029	4.772	0.807
40	N1_Block_2	299	1.5	0.814	2.247	0.998	1093.450	7.719	0.842
41	N2_Block_2	253	1.4	1.201	1.563	0.973	1144.450	7.617	0.859
42	N3_Block_2	247	1.5	1.126	2.631	1.071	1148.990	7.112	0.860
43	N4_Block_2	291	1.6	1.328	3.066	1.175	1120.800	7.493	0.884
44	N5_Block_2	299	1.5	1.002	2.244	1.122	1173.260	7.284	0.854
45	N6_Block_2	237	2.1	1.216	3.137	1.295	1129.750	7.157	0.885
46	N7_Block_2	287	1.6	1.004	2.094	1.433	1116.950	7.411	0.836
47	N8_Block_2	280	1.7	1.154	2.734	1.106	1023.480	7.560	0.893
48	N9_Block_2	310	1.7	1.002	2.666	0.976	1058.000	7.355	0.854
49	N10_Block_2	215	1.8	1.409	2.829	1.463	1190.750	7.317	0.878
50	N11_Block_2	280	1.8	1.258	2.632	1.145	1083.450	7.446	0.893
51	N12_Block_2	294	2.1	0.839	1.911	1.539	1075.630	7.303	0.864
52	N13_Block_2	254	1.5	1.104	2.640	1.137	1040.200	7.304	0.861
53	N14_Block_2	350	1.4						
54	N15_Block_2	322	1.4	1.169	2.597	1.299	1109.020	7.249	0.871
55	N16_Block_2	287	1.4	1.253	2.576	1.294	1083.760	7.425	0.879
56	N17_Block_2	338	1.7	0.946	2.533	1.138	1065.920	7.230	0.829
57	N18_Block_2	265	1.2	0.926	2.589	1.287	1099.130	7.254	0.853
58	N19_Block_2	295	1.4	1.173	2.266	1.393	1109.840	7.493	0.835
59	N20_Block_2	311	1.7	1.074	1.983	0.950	1104.510	7.614	0.854
60	N21_Block_2	326	1.6	1.211	2.104	1.168	1139.900	7.548	0.858
61	N22_Block_2	266	1.7	1.349	2.175	1.084	1168.030	7.274	0.845
62	N23_Block_2	301	1.8	1.184	2.129	1.535	1143.270	7.289	0.857
63	N24_Block_2	245	1.5	1.266	1.686	1.220	1093.970	7.842	0.865
64	N25_Block_2	329	1.7	1.072	2.035	1.179	1051.920	7.680	0.880
65	N26_Block_2	257	1.5	1.002	2.221	1.319	1117.600	7.449	0.845
66	N27_Block_2	275	1.6	1.203	3.032	1.423	1144.330	7.122	0.840
67	N28_Block_2	268	1.6	1.085	2.004	1.281	1107.220	7.637	0.871
68	N29_Block_2	249	1.7	1.584	2.205	1.045	1142.530	7.430	0.881
69	N30_Block_2	290	1.7	1.393	2.108	1.147	1127.800	7.336	0.871
70	N31_Block_2	387	1.5	0.899	2.260	1.600	1071.390	7.452	0.868
71	N32_Block_2	299	1.3	1.295	2.218	1.250	1064.470	7.534	0.885
72	N33_Block_2	280	1.5	1.315	2.339	1.355	1163.870	7.344	0.873
73	N34_Block_2	369	1.5	1.241	2.559	1.286	1138.500	7.394	0.884
74	N35_Block_2	290	1.4	1.130	3.211	1.011	1150.580	7.155	0.852
75	N36_Block_2	312	1.5	1.134	2.372	1.284	1090.230	7.577	0.875
76	N37_Block_2	312	1.4	1.134	2.372	1.284	1090.230	7.577	0.875
77	N38_Block_2	335	1.7	1.134	2.372	1.284	1090.230	7.577	0.875
78	N39_Block_2	324	1.6	1.134	2.372	1.284	1090.230	7.577	0.875
79	N1_Block_3	299	1.5	1.308	2.743	1.309	1454.460	9.931	0.882
80	N2_Block_3	253	1.4	1.532	1.820	1.439	1516.410	9.907	0.915
81	N3_Block_3	247	1.5	1.802	3.179	1.368	1470.560	9.756	0.901
82	N4_Block_3	291	1.6	1.974	3.743	1.666	1461.150	9.837	0.922
83	N5_Block_3	299	1.5	1.612	2.877	1.269	1584.360	9.510	0.895
84	N6_Block_3	237	2.1	1.775	3.612	1.632	1414.670	9.762	0.926
85	N7_Block_3	287	1.6	1.289	2.557	1.340	1461.750	9.951	0.874
86	N8_Block_3	280	1.7	1.816	3.448	1.315	1370.590	9.953	0.955
87	N9_Block_3	310	1.7	1.349	3.353	1.271	1397.290	9.644	0.882
88	N10_Block_3	215	1.8	1.975	3.428	1.718	1556.400	9.787	0.906

89	N11_Block_3	280	1.8	1.867	2.975	1.513	1415.510	9.918	0.921
90	N12_Block_3	294	2.1	1.614	2.470	1.330	1453.130	9.623	0.916
91	N13_Block_3	254	1.5	1.340	3.385	1.510	1436.130	9.257	0.887
92	N14_Block_3	350	1.4						
93	N15_Block_3	322	1.4	1.649	3.417	1.673	1429.310	9.762	0.922
94	N16_Block_3	287	1.4	1.812	3.320	1.764	1456.310	9.699	0.915
95	N17_Block_3	338	1.7	1.420	2.824	1.605	1410.000	9.576	0.872
96	N18_Block_3	265	1.2	1.287	3.423	1.497	1467.970	9.364	0.879
97	N19_Block_3	295	1.4	1.622	2.288	1.491	1401.910	10.330	0.904
98	N20_Block_3	311	1.7	1.393	2.516	1.253	1437.020	9.990	0.877
99	N21_Block_3	326	1.6	1.787	3.006	1.428	1550.420	9.830	0.903
100	N22_Block_3	266	1.7	2.049	2.761	1.640	1531.310	9.794	0.907
101	N23_Block_3	301	1.8	1.613	2.976	1.635	1500.010	9.614	0.898
102	N24_Block_3	245	1.5	1.718	2.177	1.465	1524.970	9.884	0.893
103	N25_Block_3	329	1.7	1.478	2.589	1.513	1362.600	10.226	0.922
104	N26_Block_3	257	1.5	1.459	2.807	1.355	1500.190	9.869	0.897
105	N27_Block_3	275	1.6	1.558	3.580	1.415	1513.620	9.145	0.876
106	N28_Block_3	268	1.6	1.617	2.553	1.555	1428.730	9.908	0.910
107	N29_Block_3	249	1.7	1.684	2.623	1.527	1468.670	9.847	0.915
108	N30_Block_3	290	1.7	1.856	2.585	1.317	1453.530	9.875	0.913
109	N31_Block_3	387	1.5	1.363	2.867	1.474	1402.070	9.907	0.895
110	N32_Block_3	299	1.3	1.726	3.062	1.585	1418.700	9.747	0.927
111	N33_Block_3	280	1.5	1.748	2.955	1.725	1524.920	9.776	0.913
112	N34_Block_3	369	1.5	1.754	3.078	1.579	1469.200	9.863	0.924
113	N35_Block_3	290	1.4	1.511	4.215	1.569	1534.290	9.528	0.885
114	N36_Block_3	312	1.5	1.638	2.889	1.559	1415.950	9.928	0.906
115	N37_Block_3	312	1.4	1.638	2.889	1.559	1415.950	9.928	0.906
116	N38_Block_3	335	1.7	1.638	2.889	1.559	1415.950	9.928	0.906
117	N39_Block_3	324	1.6	1.638	2.889	1.559	1415.950	9.928	0.906
118	N1_Block_4	299	1.5	1.529	2.896	1.512	1733.670	12.295	0.909
119	N2_Block_4	253	1.4	1.825	2.285	2.046	1689.870	12.275	0.929
120	N3_Block_4	247	1.5	1.950	3.670	1.411	1804.350	11.617	0.910
121	N4_Block_4	291	1.6	2.239	4.194	1.634	1690.250	12.459	0.948
122	N5_Block_4	299	1.5	2.138	3.487	1.671	1843.910	11.964	0.923
123	N6_Block_4	237	2.1	1.982	4.270	1.859	1706.060	12.071	0.928
124	N7_Block_4	287	1.6	1.653	2.758	1.031	1747.310	12.090	0.889
125	N8_Block_4	280	1.7	1.775	3.607	1.547	1612.610	12.472	0.962
126	N9_Block_4	310	1.7	1.813	3.130	1.784	1690.550	11.885	0.900
127	N10_Block_4	215	1.8	2.374	3.711	1.749	1827.370	12.412	0.940
128	N11_Block_4	280	1.8	2.160	3.380	1.721	1637.160	12.367	0.943
129	N12_Block_4	294	2.1	2.061	2.677	2.457	1746.160	12.185	0.928
130	N13_Block_4	254	1.5	1.554	3.640	1.581	1652.970	11.768	0.923
131	N14_Block_4	350	1.4						
132	N15_Block_4	322	1.4	2.224	3.932	1.962	1715.330	12.114	0.919
133	N16_Block_4	287	1.4	1.912	3.688	2.067	1695.300	12.040	0.930
134	N17_Block_4	338	1.7	1.698	3.826	1.740	1720.710	11.804	0.906
135	N18_Block_4	265	1.2	1.722	3.455	1.886	1762.890	11.838	0.907
136	N19_Block_4	295	1.4	1.858	2.557	1.894	1711.480	12.594	0.916
137	N20_Block_4	311	1.7	1.902	2.731	1.608	1679.940	12.655	0.903
138	N21_Block_4	326	1.6	2.235	3.201	1.787	1790.720	12.302	0.926

139	N22_Block_4	266	1.7	2.406	3.245	1.871	1812.720	12.075	0.920
140	N23_Block_4	301	1.8	2.163	3.256	1.798	1749.660	12.203	0.920
141	N24_Block_4	245	1.5	2.002	2.548	1.782	1784.080	12.566	0.921
142	N25_Block_4	329	1.7	1.796	2.859	1.673	1635.410	12.355	0.946
143	N26_Block_4	257	1.5	1.526	2.682	1.498	1719.410	12.560	0.907
144	N27_Block_4	275	1.6	1.836	3.809	1.484	1741.800	11.864	0.891
145	N28_Block_4	268	1.6	2.215	2.915	1.713	1664.910	12.461	0.930
146	N29_Block_4	249	1.7	1.737	2.965	1.832	1694.890	12.477	0.935
147	N30_Block_4	290	1.7	2.393	2.804	1.468	1677.590	12.354	0.940
148	N31_Block_4	387	1.5	1.692	3.419	1.524	1644.280	12.174	0.926
149	N32_Block_4	299	1.3	1.821	3.339	1.847	1633.360	12.295	0.938
150	N33_Block_4	280	1.5	2.300	3.209	1.862	1731.510	12.337	0.941
151	N34_Block_4	369	1.5	2.209	3.482	1.827	1754.450	12.237	0.939
152	N35_Block_4	290	1.4	1.805	4.454	1.702	1843.630	11.653	0.904
153	N36_Block_4	312	1.5	2.041	3.092	1.753	1662.880	12.386	0.932
154	N37_Block_4	312	1.4	2.041	3.092	1.753	1662.880	12.386	0.932
155	N38_Block_4	335	1.7	2.041	3.092	1.753	1662.880	12.386	0.932
156	N39_Block_4	324	1.6	2.041	3.092	1.753	1662.880	12.386	0.932
157	N1_Block_5	299	1.5	1.662	3.358	1.611	1960.130	14.618	0.910
158	N2_Block_5	253	1.4	1.952	2.364	2.046	1995.290	14.442	0.940
159	N3_Block_5	247	1.5	2.344	3.750	1.577	2032.980	14.400	0.931
160	N4_Block_5	291	1.6	2.561	4.586	2.075	1846.250	14.845	0.971
161	N5_Block_5	299	1.5	2.323	3.705	1.793	2216.710	13.730	0.928
162	N6_Block_5	237	2.1	2.535	4.565	2.121	1868.930	14.877	0.950
163	N7_Block_5	287	1.6	2.006	3.215	1.463	2064.780	14.664	0.917
164	N8_Block_5	280	1.7	2.090	3.966	1.699	1697.960	15.146	0.966
165	N9_Block_5	310	1.7	2.082	3.463	1.676	1862.300	14.633	0.924
166	N10_Block_5	215	1.8	2.512	4.455	1.911	1950.690	14.882	0.943
167	N11_Block_5	280	1.8	2.288	3.365	1.896	1827.660	14.820	0.956
168	N12_Block_5	294	2.1	2.430	2.577	2.067	1971.760	14.695	0.946
169	N13_Block_5	254	1.5	1.713	3.843	1.731	1846.820	14.477	0.922
170	N14_Block_5	350	1.4						
171	N15_Block_5	322	1.4	2.237	4.101	2.094	1906.870	14.657	0.948
172	N16_Block_5	287	1.4	2.355	3.625	2.202	1868.030	14.660	0.943
173	N17_Block_5	338	1.7	2.022	3.785	1.989	1939.450	14.249	0.930
174	N18_Block_5	265	1.2	2.090	3.715	2.142	1930.680	14.523	0.925
175	N19_Block_5	295	1.4	1.770	2.967	1.998	1980.540	14.876	0.943
176	N20_Block_5	311	1.7	2.181	2.932	1.974	1897.150	15.041	0.924
177	N21_Block_5	326	1.6	2.735	3.303	2.007	2044.830	14.833	0.945
178	N22_Block_5	266	1.7	2.652	3.499	1.892	2064.010	14.479	0.935
179	N23_Block_5	301	1.8	2.488	3.594	2.067	2007.200	14.344	0.932
180	N24_Block_5	245	1.5	2.237	3.384	2.333	2096.870	14.166	0.946
181	N25_Block_5	329	1.7	1.666	3.032	1.779	1819.490	14.900	0.945
182	N26_Block_5	257	1.5	1.986	3.385	1.623	1954.720	14.654	0.929
183	N27_Block_5	275	1.6	1.891	4.029	1.686	1958.890	14.208	0.914
184	N28_Block_5	268	1.6	2.534	2.986	1.894	1851.190	14.932	0.953
185	N29_Block_5	249	1.7	2.468	3.544	1.713	1910.580	14.712	0.953
186	N30_Block_5	290	1.7	2.557	3.061	1.761	1880.000	14.791	0.946
187	N31_Block_5	387	1.5	1.714	3.419	1.749	1836.900	14.555	0.938
188	N32_Block_5	299	1.3	2.387	3.535	1.916	1797.570	14.867	0.945

189	N33_Block_5	280	1.5	2.472	3.755	2.042	2009.500	14.765	0.951
190	N34_Block_5	369	1.5	2.385	3.825	1.855	1930.390	14.722	0.951
191	N35_Block_5	290	1.4	2.007	3.996	2.041	2060.120	14.256	0.929
192	N36_Block_5	312	1.5	1.885	3.548	1.901	1849.540	15.035	0.940
193	N37_Block_5	312	1.4	1.885	3.548	1.901	1849.540	15.035	0.940
194	N38_Block_5	335	1.7	1.885	3.548	1.901	1849.540	15.035	0.940
195	N39_Block_5	324	1.6	1.885	3.548	1.901	1849.540	15.035	0.940

Xa- average particle size (μm); TS – tensile strength (MPa); DS- detachment stress (MPa); ES- ejection stress (MPa); WoC- work of compression (J); ER- elastic recovery (%); SF- solid fraction; empty cell – missing experimental data.

Table S3. Estimated compression parameters.

Primary ID	k_{Heckel}	Py	Kb	d	g
N1	0.007	153.307	11.136	0.016	0.921
N2	0.009	115.539	8.987	0.033	0.811
N3	0.008	128.846	11.038	0.014	1.016
N4	0.013	79.6007	8.556	0.031	0.862
N5	0.008	118.134	8.559	0.024	0.893
N6	0.009	117.552	10.213	0.03	0.855
N7	0.007	144.481	9.86	0.021	0.885
N8	0.012	83.5694	8.543	0.03	0.833
N9	0.007	145.522	10.912	0.02	0.905
N10	0.009	111.621	9.71	0.03	0.879
N11	0.01	103.305	9.752	0.03	0.859
N12	0.009	107.421	12.209	0.006	1.183
N13	0.007	141.316	8.608	0.046	0.714
N15	0.009	112.551	9.148	0.026	0.881
N16	0.009	116.346	9.062	0.034	0.826
N17	0.008	117.904	9.507	0.015	0.965
N18	0.007	135.480	10.567	0.016	0.947
N19	0.01	104.517	6.765	0.049	0.725
N20	0.007	141.934	10.78	0.021	0.904
N21	0.009	105.509	9.082	0.024	0.917
N22	0.009	116.369	8.266	0.042	0.812
N23	0.008	119.756	10.485	0.013	1.029
N24	0.009	108.825	7.592	0.056	0.723
N25	0.009	106.853	8.036	0.042	0.743
N26	0.008	130.221	8.263	0.041	0.747
N27	0.007	152.727	8.847	0.043	0.756
N28	0.009	105.526	9.628	0.027	0.877
N29	0.009	107.714	8.338	0.058	0.718
N30	0.009	111.545	10.108	0.031	0.87
N31	0.008	121.241	10.376	0.018	0.901
N32	0.009	116.552	9.567	0.029	0.851
N33	0.01	98.7365	8.4	0.033	0.845
N34	0.009	109.073	9.563	0.033	0.836
N35	0.008	128.589	8.436	0.036	0.789
N36	0.008	124.637	9.183	0.044	0.754
N37	0.008	121.645	9.201	0.043	0.753
N38	0.008	123.487	9.182	0.044	0.760
N39	0.008	124.574	9.173	0.045	0.755

Table S4. Influence of raw material and process related factors on compression parameters.

Table .	kH	Py	kb	d	g
Constant	3.54×10^{-23}	2.50×10^{-23}	2.05×10^{-27}	1.06×10^{-15}	1.90×10^{-27}
Spray rate	2.58×10^{-1}	3.42×10^{-1}	1.11×10^{-1}	9.19×10^{-2}	3.29E $\times 10^{-1}$
Atomizing pressure	1.73E $\times 10^{-1}$	2.04×10^{-1}	6.21×10^{-1}	3.70×10^{-1}	6.46E $\times 10^{-1}$
PAR					
PAR A	5.78×10^{-1}	3.61×10^{-1}	3.39×10^{-1}	6.47×10^{-1}	1.04×10^{-1}
PAR B	3.75×10^{-1}	4.12×10^{-1}	8.46×10^{-1}	6.09×10^{-1}	3.49×10^{-1}
PAR C	7.69×10^{-1}	8.78×10^{-1}	4.17×10^{-1}	9.80×10^{-1}	4.15×10^{-1}
IBU					
IBU A	7.55×10^{-1}	6.37×10^{-1}	4.76×10^{-1}	7.72×10^{-1}	4.20×10^{-1}
IBU B	6.72×10^{-1}	8.76×10^{-1}	4.91×10^{-1}	9.54×10^{-1}	9.98×10^{-1}
IBU C	4.70×10^{-1}	5.34×10^{-1}	9.71×10^{-1}	8.17×10^{-1}	4.21×10^{-1}
MCC					
MCC A	7.63×10^{-1}	9.30×10^{-1}	2.05×10^{-2}	3.39×10^{-3}	1.32×10^{-2}
MCC B	7.63×10^{-1}	9.30×10^{-1}	2.05×10^{-2}	3.39×10^{-3}	1.32×10^{-2}
Spray*Pres	3.45×10^{-1}	6.01×10^{-1}	2.32×10^{-1}	1.19×10^{-1}	1.24×10^{-1}
Spray*Par					
Spray*PAR A	9.07×10^{-1}	9.70×10^{-1}	4.51×10^{-1}	6.38×10^{-1}	5.60×10^{-1}
Spray *PAR B	5.56×10^{-1}	6.48×10^{-1}	5.36×10^{-1}	7.01×10^{-1}	7.14×10^{-1}
Spray *PAR C	5.03×10^{-1}	6.92×10^{-1}	8.91×10^{-1}	9.31×10^{-1}	8.33×10^{-1}
Spray *Ibu					
Spray *IBU A	3.56×10^{-1}	2.68×10^{-1}	9.17×10^{-2}	2.51×10^{-1}	3.00×10^{-1}
Spray *IBU B	3.86×10^{-1}	3.92×10^{-1}	2.29×10^{-1}	4.69×10^{-1}	3.68×10^{-1}
Spray *IBU C	9.84×10^{-1}	8.41×10^{-1}	6.76×10^{-1}	7.16×10^{-1}	9.24×10^{-1}
Spray *MCC					
Spray *MCC A	3.25×10^{-1}	5.80×10^{-1}	3.75×10^{-2}	5.56×10^{-2}	6.27×10^{-2}
Spray *MCC B	3.25×10^{-1}	5.80×10^{-1}	3.75×10^{-2}	5.56×10^{-2}	6.27×10^{-2}

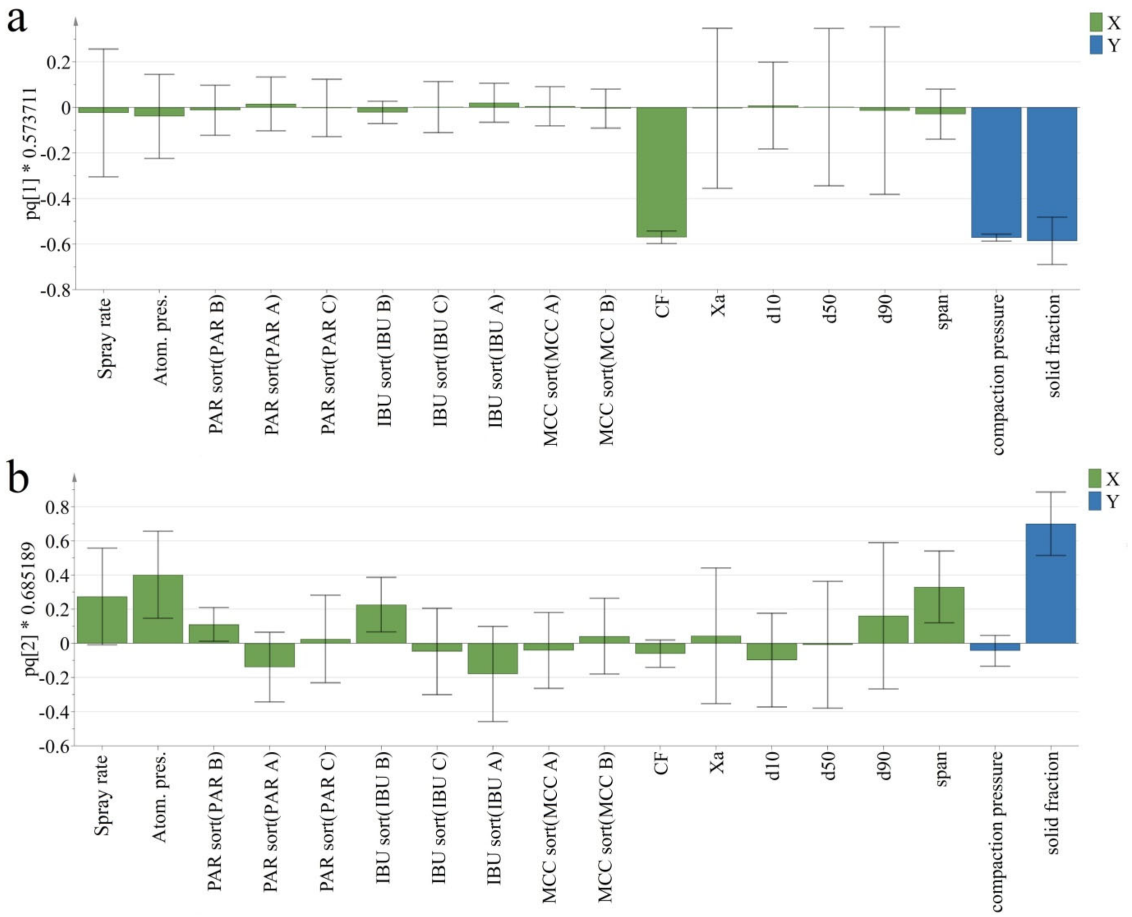

Figure S1. pq loading plot for the first and second predictive component of the O2PLS model fitted for Compressibility.

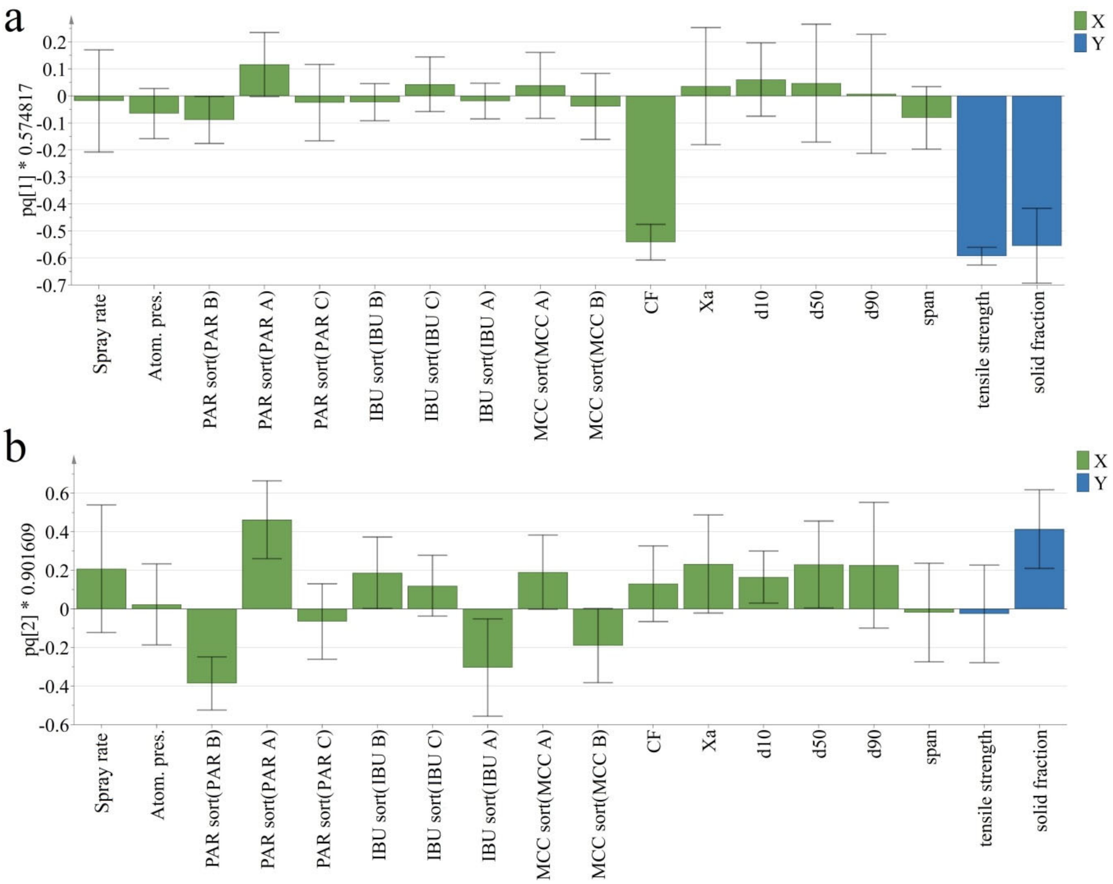

Figure S2. pq loading plot for the first and second predictive component of the O2PLS model fitted for Compactability.

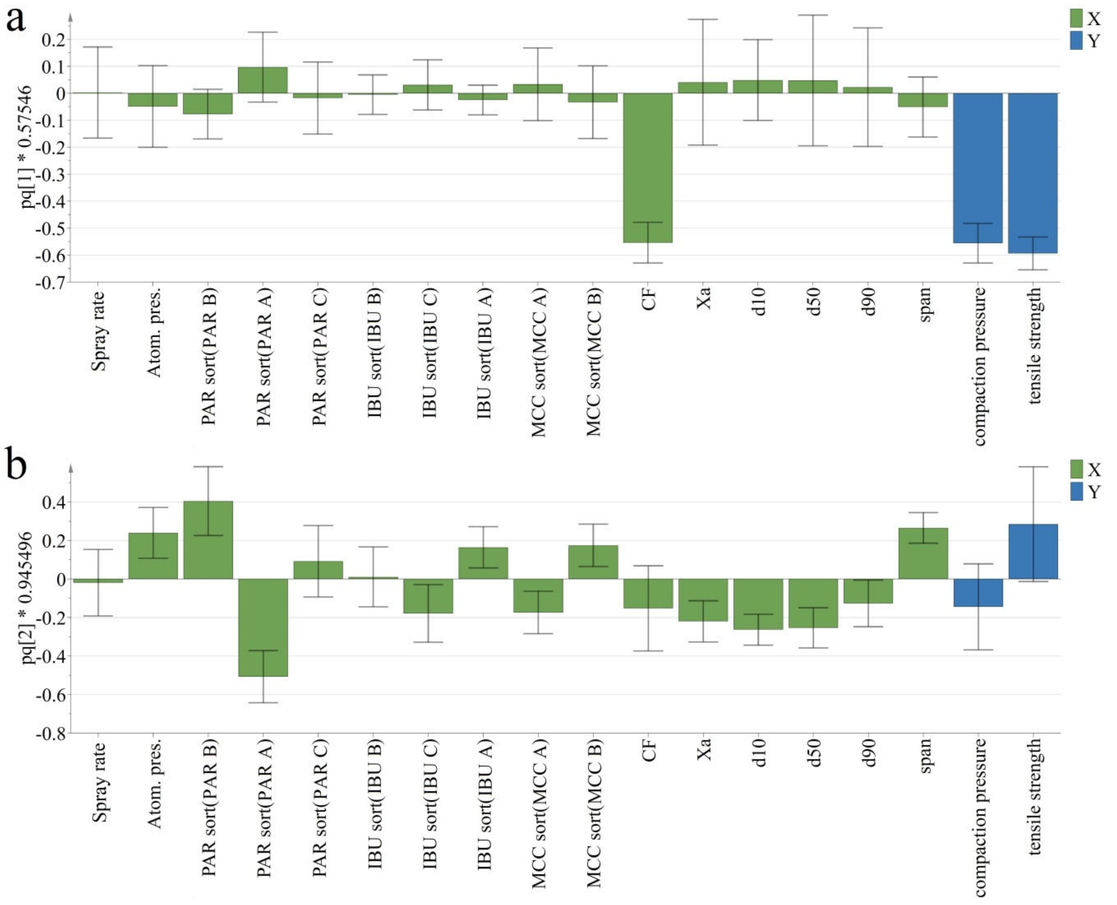

Figure S3. pq loading plot for the first and second predictive component of the O2PLS model fitted for Tabletability.
